# Slip or fallacy? Effects of error severity on own and observed pitch error processing in pianists

**DOI:** 10.3758/s13415-023-01097-1

**Published:** 2023-05-17

**Authors:** Christine Albrecht, Christian Bellebaum

**Affiliations:** Institute of Experimental Psychology, Heinrich Heine University Düsseldorf, Universitätsstraße 1, building 23.03, room number 00.89, 40225 Düsseldorf, Germany

**Keywords:** Error severity, Action monitoring, Observed action monitoring, ERN, oERN

## Abstract

Errors elicit a negative, mediofrontal, event-related potential (ERP), for both own errors (error-related negativity; ERN) and observed errors (here referred to as observer mediofrontal negativity; oMN). It is unclear, however, if the action-monitoring system codes action valence as an all-or-nothing phenomenon or if the system differentiates between errors of different severity. We investigated this question by recording electroencephalography (EEG) data of pianists playing themselves (Experiment 1) or watching others playing (Experiment 2). Piano pieces designed to elicit large errors were used. While active participants’ ERN amplitudes differed between small and large errors, observers’ oMN amplitudes did not. The different pattern in the two groups of participants was confirmed in an exploratory analysis comparing ERN and oMN directly. We suspect that both prediction and action mismatches can be coded in action monitoring systems, depending on the task, and a need-to-adapt signal is sent whenever mismatches happen to indicate the magnitude of the needed adaptation.

**Supplementary Information:**

The online version contains supplementary material available at 10.3758/s13415-023-01097-1.

During the past 30 years, researchers have intensely investigated the neural correlates of error processing (Falkenstein et al., 1991; Gehring et al., 1993; Jessup et al., 2010; Ullsperger et al., 2014). Contrasting errors versus correct actions showed that error processing involves several areas at the medial wall of the prefrontal cortex (this region will be referred to as medial prefrontal cortex or mPFC), including the anterior cingulate cortex (ACC; Debener et al., 2005; Ullsperger et al., 2014). An event-related potential (ERP) component investigated in the context of error processing is the error-related negativity (ERN), a negative-going frontocentral deflection that peaks around 100 ms after an erroneous response (Falkenstein et al., 1991; Falkenstein et al., 2000; Gehring et al., 1993, Gehring et al., 2012; Holroyd and Coles, 2002). The ERN appears to be generated in the mPFC, probably the ACC (Debener et al., 2005; Dehaene et al., 1994; Ridderinkhof et al., 2004; Taylor et al., 2007).

Not all researchers agree that the mPFC is primarily involved in error processing. Thus, it also has been questioned whether the ERN reflects error processing per se. Apart from a conception in terms of conflict monitoring (Botvinick et al., 2001; Carter et al., 1998; Yeung et al., 2004), Holroyd and Coles (2002) suggest that the ERN represents a reinforcement learning signal that is used to optimize performance. This signal would not only include the information if an action is right or wrong, but, in case, also the extent of the error as well as whether the event was more or less unexpected—representing a signed prediction error. The more recent predicted-response outcome model (PRO model; Alexander and Brown, 2011) states that mPFC activity reflects unexpected events, e.g., outcomes and actions, rather than errors, i.e., an unsigned prediction error (Gawlowska et al., 2018; Jessup et al., 2010; Wessel et al., 2012). Although there is initial evidence supporting the PRO model in the sense that error and surprise processing rely on similar neural mechanisms (Jessup et al., 2010; Wessel et al., 2012), there also is reason to believe that the mPFC codes information that is particularly relevant for error processing (Hajcak et al., 2005; Maier et al., 2012; Maier and Steinhauser, 2016), which is more in line with the reinforcement learning theory (Holroyd and Coles, 2002).

Both the reinforcement learning theory (Holroyd and Coles, 2002) and the PRO model (Alexander and Brown, 2011) imply that an ACC-driven response-locked neural signal is sensitive to (prediction) error size. Indeed, previous empirical studies have found that ERN amplitude can vary in different contexts and conditions. For example, ERN amplitude is enhanced when errors are particularly significant or participants are more motivated (Ganushchak and Schiller, 2008; Gehring et al., 1993; Hajcak et al., 2005). The actions themselves in these studies were, however, always classified in a binary fashion as either right or wrong. Action valence can vary more gradually than just distinguishing right versus wrong. In sports, music or many other motor-cognitive tasks, people can diverge from the correct movement on a scale from “perfect” to “completely wrong.” In everyday language, we use terms such as *slip* or *fallacy*, which also suggests that we distinguish between errors of different severity. The ACC receives input from both motor and cognitive brain areas and is supposedly involved in the planning and regulation of behavior (Devinsky et al., 1995), making it a crossroad for correction and adaptation (Holroyd and Coles, 2002). For this function, the system needs to know how much adaptation is needed: for example, when a pianist hits a key one or two notes amiss and must adapt their hand position within milliseconds to hit the next note. Taking into account the function of the ACC, the variability of ERN amplitudes in different contexts (Ganushchak and Schiller, 2008; Gehring et al., 1993; Hajcak et al., 2005) and the early processing needed for error (severity) detection in order to adapt behavior, it is conceivable that error severity is processed early after error commission in the time window of the ERN. We thus assume that the ERN as a fast indicator of information related to error processing codes action valence on a spectrum and not as an all-or-nothing phenomenon, thus reflecting error severity. Although the PRO model (Alexander and Brown, 2011) assumes effects of error severity in the sense of the magnitude of a prediction error, the previously mentioned findings would support the view that the mPFC/ACC is, at least partially, involved in representing performance accuracy and not entirely driven by event expectancy, as stated by the PRO model (see for example Maier and Steinhauser, 2016 for conflicting results regarding the model), which is more in line with the reinforcement learning theory (Holroyd and Coles, 2002), stating that the ERN reflects a learning signal to optimize performance.

Initial evidence supporting the assumption of a continuous encoding of error severity stems from studies comparing different types of responses yielding different error types (under-reach vs. over-reach, Murata and Katayama, 2005; hand vs. finger, Falkenstein et al., 2000; corrected vs. uncorrected, Paas et al., 2021). An effect of error size has been described in two paradigms in which wrong actions in either one (single error) or two (double error) dimensions were possible (Bernstein et al., 1995; Maier et al., 2008, 2012; Maier and Steinhauser, 2016): double errors led to significantly larger ERN amplitudes than single errors. These results, however, also may be explained by two parallel action monitoring processes for both dimensions, each coding accuracy in a binary fashion, that add up to an increased ERN. It has yet to be investigated whether different degrees of deviations from the aspired action indeed lead to correspondingly increased neural responses in action monitoring regions.

The reinforcement learning theory (Holroyd and Coles, 2002) implies that the ACC acts as a motor control unit, and therefore, an ERN should only occur when the person has acted in some way. In contrast, the processing of observed actions has been suggested to involve similar brain areas as the processing of self-actions, such as the mPFC, specifically the ACC (Yoshida et al., 2012; Koban and Pourtois, 2014) and presupplementary and supplementary motor areas (Scangos et al., 2013), with additional activity, inter alia, in the superior temporal sulcus (Ninomiya et al., 2018), inferior frontal gyrus (Shane et al., 2008), and anterior insula (Cracco et al., 2016; Koban and Pourtois, 2014). Accordingly, observed errors have been reported to elicit an ERP component corresponding to the ERN, the observer error-related negativity (oERN) at frontocentral sites (Bates et al., 2005; de Bruijn and von Rhein, 2012; Miltner et al., 2004; van Schie et al., 2004). Source localization suggests the origin of the oERN also in the mPFC (van Schie et al., 2004), probably in the ACC (Miltner et al., 2004). Compared with the ERN, the oERN displays smaller amplitudes and peaks later relative to the eliciting event, which is an observed action and thus a visual stimulus rather than an own motor response, with the latency depending on the task (Bates et al., 2005; de Bruijn and von Rhein, 2012; van Schie et al., 2004). Research in observed error processing, as in own error processing, has mostly focused on binary response classifications in terms of accuracy (Bates et al., 2005; de Bruijn & von Rhein, 2012; Kobza and Bellebaum, 2013). Recent evidence from our lab indicated, however, that observed responses are processed primarily based on their expectancy and not their accuracy (Albrecht and Bellebaum, 2021a, 2021b; Desmet et al., 2014), which might lead to differences compared to active responding with respect to effects of error severity. For observed action monitoring, the PRO model (Alexander and Brown, 2011) seems to fit empirical results better than the reinforcement learning theory (Holroyd and Coles, 2002).

To investigate whether the ERN does indeed reflect a signal for action adaptation, and to compare effects on own and observed action monitoring, we investigated the effects of error severity in both an active and observation condition. So-called sequential tasks, such as typing or playing the piano (Herrojo Ruiz et al., 2009; Kalfaoğlu et al., 2018; Maidhof et al., 2009; Maidhof et al., 2013; Paas et al., 2021), appear to be particularly suitable to study error severity effects. In these tasks, errors are frequent and participants stay seated while performing a (highly practiced) everyday motor task that is ecologically valid and not dependent on feedback (Herrojo Ruiz et al., 2009). Typically, the ERN occurs 20-100 ms before the response in sequential tasks (Herrojo Ruiz et al., 2009; Kalfaoğlu et al., 2018; Maidhof et al., 2009; Paas et al., 2021) and thus earlier than in tasks involving a single response (Falkenstein et al., 1991; Gehring et al., 1993). Maidhof et al. (2013) showed that potential errors are noticed earlier with regard to the registered keypress (probably due to earlier movement onset compared with nonsequential tasks), and earlier error registration is associated with shorter ERN latencies (Di Gregorio et al., 2022). Furthermore, error monitoring and error severity processing are especially important for adaptation during sequential tasks.

In the present study, we thus conducted two experiments with pianists. In Experiment 1, participants played piano pieces which included frequent changes of hand positions, thereby provoking small and large errors. While participants played, both EEG and behavioral data were assessed. Videos recorded during Experiment 1 served as stimuli for Experiment 2, in which participants watched videos of other pianists performing while EEG data were assessed in the observers. With these experiments we aimed to investigate two main questions: First, are ERN amplitudes enhanced for larger compared to smaller errors? Second, is a similar effect found also for observed errors?

## Experiment 1

In Experiment 1, we studied effects of error severity on error processing during active piano playing. Apart from the neural processing of errors, the piano-playing paradigm allows investigation of relevant behavioral variables. First, post-event reaction times can be assessed. A relative slowing of reaction times after errors is a well-studied phenomenon (Rabbitt, 1966, 1969), possibly linked to an attentional shift towards the error (or unexpected event), resulting in an attention reorienting process back to the task that underlies the longer reaction times (Notebaert et al., 2009; Núñez Castellar et al., 2010). Post-error slowing is presumably modulated by activity in the ACC (Danielmeier et al., 2011; Debener et al., 2005; Fu et al., 2019), but findings on the relationship between ERN and post-error slowing are mixed (Chang et al., 2014; Debener et al., 2005; Gehring et al., 1993; Hajcak et al., 2003). Possibly, some factors influence post-error slowing and the ERN differently (such as expertise, Jentzsch et al., 2014, or error awareness, Nieuwenhuis et al., 2001), leading to a dissociation in respective tasks. Post-error slowing also has been observed in piano-playing tasks (Herrojo Ruiz et al., 2009; Paas et al., 2021). A second variable of interest is keypress volume (assessed as velocity), as error notes were played significantly more quietly than correct notes in previous piano-playing studies (Herrojo Ruiz et al., 2009; Maidhof et al., 2009; Maidhof et al., 2013; Paas et al., 2021). Because larger errors might lead to a larger focus of attention on the error, enhanced post-error slowing was expected for large compared to small errors. Additionally, quieter keypress volumes of error keypresses compared with correct keypresses were expected, but as the processes behind the volume reduction are not yet established, we refrain from predicting differences between small and large errors regarding volume.

### Method

#### Participants

We recruited experienced pianists to take part in the study via social media, person-to-person recruiting, and flyers distributed at the university, music conservatory, and music schools. Because the pieces included large steps between keys to induce errors and the pieces were thus difficult to learn, we suggested a minimum experience of 1,500 hours spent with the instrument, although participants were allowed to take part with less experience if they were able to play the pieces fluently. We aimed for a sample size of at least 20 participants, because this sample size seems to be adequate for sequential tasks (Herrojo-Ruiz et al., 2009; Kalfaoğlu et al., 2018; Maidhof et al., 2013). Expecting a 30% dropout-rate for fulfilling one or more exclusion criteria or due to technical problems, we originally recruited 30 participants. Of these, five were excluded due to previous neurological or psychological diseases, so data from 25 participants were recorded. Of these, one had to be excluded due to technical problems during data acquisition. Another three were excluded because they made less than ten large errors, which was especially problematic for the analysis of the ERP data (see below). The remaining sample of 21 participants consisted of 12 cis-gender women and 9 cis-gender men between 17 and 34 years (mean [*M*] = 23.1 years, standard deviation [*SD*] = 4.2 years). Twenty of them were right-handed, one person was left-handed. Please note that pianists usually play melodies with their right hand and accompaniment with their left hand, regardless of handedness, so left-handed participants should be able to perform the task as well as right-handed participants, as was the case for the left-handed participant that took part in our study. All participants reported no previous neurological or psychiatric illnesses and no intake of medication that affected the nervous system. All participants took part voluntarily. The study is in compliance with the declaration of Helsinki and was approved by the ethics committee of the Faculty of Mathematics and Natural Sciences at Heinrich-Heine-University, Düsseldorf.

#### Material

We designed six pieces to be played with only the right hand. All pieces consisted of 96 sixteenth notes in 6 bars and ended with a seventh bar that consisted of a single whole note. To keep the physical distance between played keys constant, all pieces were written in C major and thus only played on white keys. The pieces kept to a general harmonic structure and the highest notes played could be interpreted as a melody, the remaining notes as accompaniment. The pieces were designed to require large hand movements to induce errors. The lowest key throughout the pieces was E3, the highest key was A5. Consecutive notes could differ between 1 and 10 white keys; the average difference was 4.98 white keys (*SD* = 1.88 keys). The pieces were written in MuseScore 3 (version 3.6.2, MuseScore BVBA, 2021). They are included in the Supplementary Material (Figure S1).

An automatically created recording was generated for each of the pieces (created with MuseScore 3, version 3.6.2, MuseScore BVBA, 2021) in which the melody parts of the pieces were pronounced. In the recording, pieces were played at 60 beats per minute (one beat = one quarter note), and tempo at the top of the score notation also was stated as 60 quarter notes per minute.

#### Experimental task and setup

The pieces as well as the recording were sent to each participant 2 weeks before testing. Participants were instructed to study the pieces in the next 14 days. They were told that they should be able to play the pieces with the right hand quite fluently but that they should not strive for perfect sound and that occasional errors during play were acceptable. Participants also were told to practice in whatever tempo they felt comfortable. They were given an instruction to practice approximately 15 minutes a day (distributed as they saw fit). According to self-reports, the participants practiced the pieces 204.1 minutes on average (*SD* = 188.9 minutes, 45-840 minutes).

**Fig. 1:**
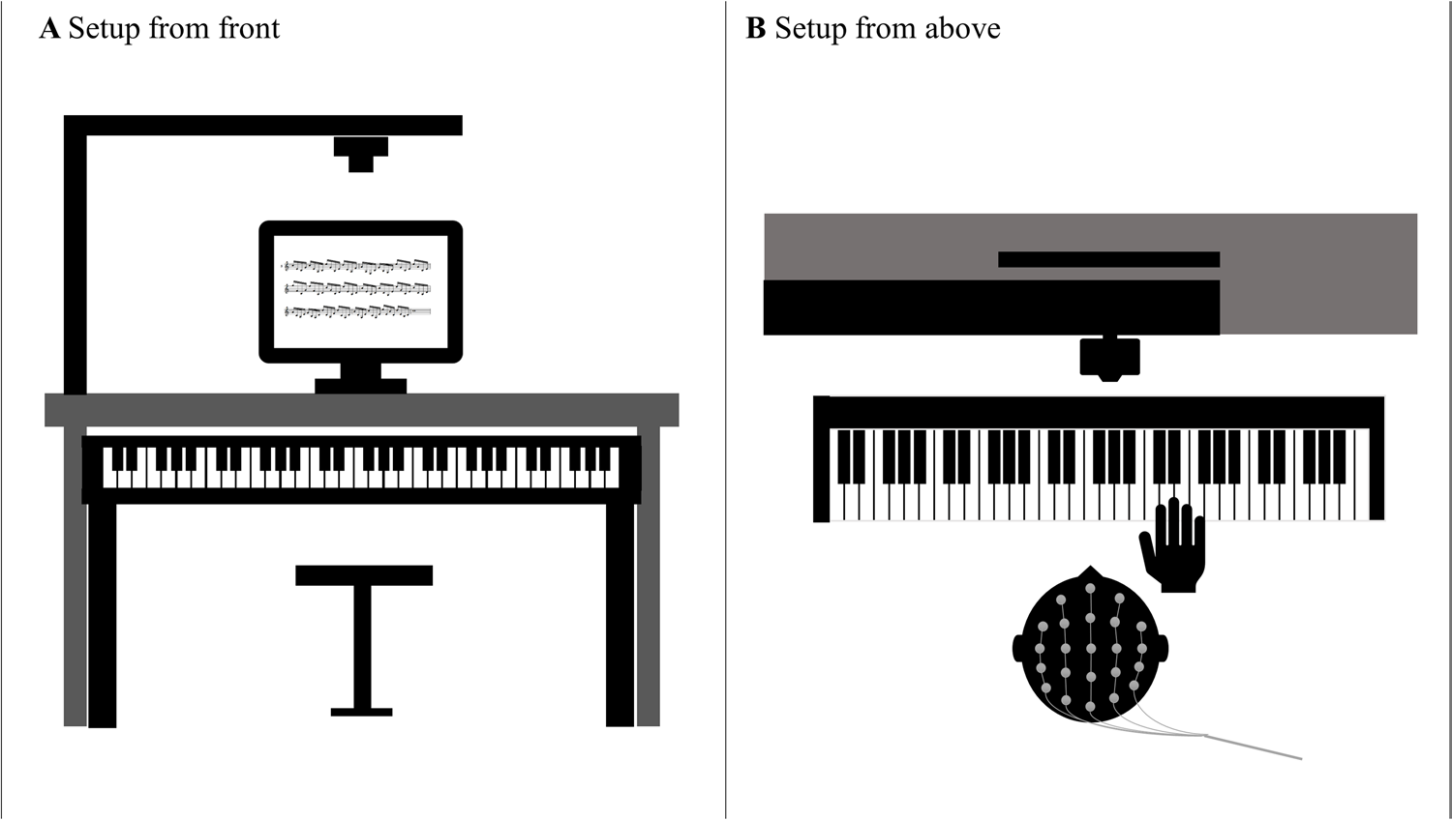
Setup of Experiment 1

For data acquisition during the experiment, participants used a digital piano (Casio LK-S450 for most participants, two participants used a Yamaha YDP-144 R Arius). During the experiment, the keyboard was set on mute, so that participants could not hear themselves play. The piano was positioned in front of a desktop monitor (1,920 x 1,080 px) that served for visual stimulation. Participants could navigate through the experiment with their left hand and the lowest note on the keyboard. A Logitech BRIO webcam was connected to an additional laptop for recording the participants’ hand from above during play for the videos used in Experiment 2. A picture of the setup can be seen in Fig. 1. We recorded the Musical Instrument Digital Interface (MIDI) information of the played segments on the experiment computer. MIDI refers to the signal used by digital instruments to generate and communicate tones including note on- and offset, key and velocity (in piano playing, this corresponds to volume). Stimulus presentation, EEG trigger timing and MIDI recording was controlled with Python 3.7.5 using the packages psychopy (version 3.2.3; Peirce et al., 2019) and mido (version 1.2.9, Ole Martin Bjørndalen 2021, mido.readthedocs.io).

**Fig. 2:**
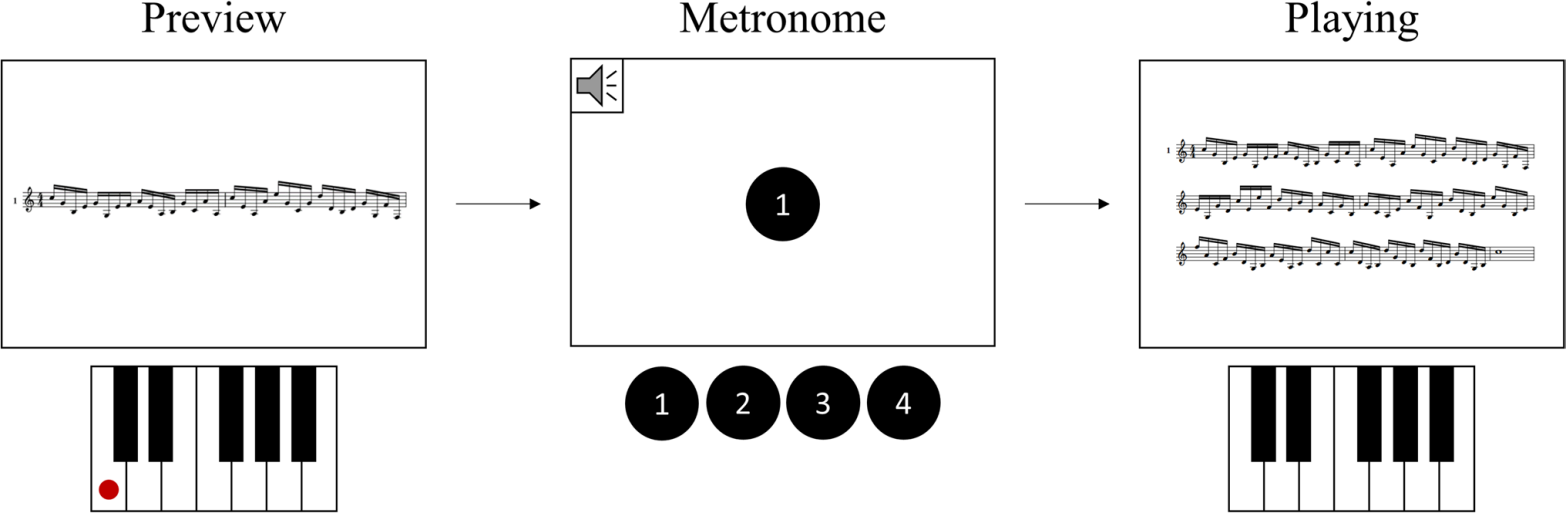
Sequence structure of Experiment 1

The experiment consisted of 60 sequences in total, 10 for each piece. Each sequence started with a score notation preview of the piece that was to be played (a picture of the first two bars, i.e. the first line, of the respective piece score notation, including the piece number). Participants could then start the recording which began with 4 metronome beats (1,000-hz beeps) accompanied by the numbers 1 to 4 displayed on the screen. Subsequently, the score notation of the whole piece was displayed on the screen to allow participants to play from sheet. After they finished playing the piece, participants ended the recording and proceeded to the next sequence. A display of the sequence structure can be seen in Fig. 2.

Before the experiment, participants were asked in what tempo they had practiced the pieces. Accordingly, the metronome beats were set for each participant individually to a tempo slightly faster than the tempo in which they had practiced to increase difficulty. Participants were instructed to start playing right after the last metronome beat had been presented. They were further asked to keep to one tempo (loosely that of the metronome) during each sequence and to put emphasis on playing fluently, even if that meant making errors.

The 60 sequences were preceded by 3 practice sequences in which participants could get to know the procedure of a sequence, but in which they were shown a mock preview and no actual score notation during play. They were instructed to get familiar with the instrument and the procedure during these practice sequences, and to play whatever came to their mind.

#### Assessment of expertise

As a measure of Piano Playing Expertise we assessed the experience participants had with their instrument, because a certain level of experience was defined as inclusion criterion (see above). Expertise was defined as total hours spent with the instrument, calculated by multiplying the self-reported number of years of piano experience with the self-reported average hours of practice per week times 52 (number of weeks per year).

#### EEG recording

We recorded EEG signals at a 1,000-Hz sampling rate with a 32-channel actiCap electrode cap (ActiCAP; Brain Products GmbH, Germany) with the software Brain Vision Recorder (version 1.20, Brain Products, Munich, Germany). The active silver/silver-chloride electrodes were attached according to the 10-20 system on 29 scalp sites, i.e., FCz (which was used as online reference), F7, F3, Fz, F4, F8, FC5, FC1, FC2, FC6, T7, C3, Cz, C4, T8, CP5, CP1, CP2, CP6, P7, P3, Pz, P4, P8, PO9, P1, Pz, P2, and PO10. Additionally, we recorded the signal from both mastoids to use as offline reference. The ground electrode was placed at AFz. For electrooculogram (EOG) data, we placed two horizontal EOG (hEOG) electrodes at F9 and F10, respectively, and two vertical EOG (vEOG) electrodes at Fp2 and below the right eye. All impedances were kept below 10 kΩ.

An EEG marker was sent every fifth keystroke to avoid a possible overlap of markers (Maidhof et al., 2009). The MIDI data allowed offline determination of markers for the remaining keystrokes. We conducted a pilot test for a possible delay between key press and marker by using a Tektronix TDS 210 oscilloscope. Key presses are transformed to audio signals by the digital instrument in real-time. In the test, we therefore compared onset times between the audio and marker signal. The markers were sent consistently 1.6 ms before tone onset across all tests.

#### Procedure

Participants received the piano pieces 2 weeks before the actual experiment in the lab. After arrival, participants gave informed written consent to take part in the study and completed a demographic questionnaire and an expertise self-report measure.

Subsequently, EEG electrodes were attached to the scalp and participants started the experiment. Participants received written instructions and the experimenters were present during three practice sequences for questions and further explanations. At the start of the experiment, recordings of video, MIDI and EEG were started. The experiment lasted between 35 and 75 minutes, depending on the speed in which participants played. After completion of the experiment, participants received compensation in the form of either course credit or 40 €.

#### Data analyses

##### Behavioural data preprocessing and definition of event types

All following analysis steps were performed in MATLAB, version R2017b (Mathworks, Natick, MA). We employed the MATLAB MIDI Toolbox (Eerola and Toiviainen, 2004) and a dynamic score matcher algorithm created by Large (1993; see also Palmer and van de Sande, 1993; Rankin et al., 2009) to compare the recorded MIDI signal with the correct score notation the participants had been asked to play. This procedure was used to determine the different types of trials for which ERP and behavioral data were compared (see below). The algorithm finds a so-called optimal match between two MIDI sequences and assigns every played note an attribute: match, substitution (a score notation note was replaced in the performance), addition (there was an added note in the performance that could not be matched to any notation note), and miss. All substitution events were defined as “uncorrected” errors (see also below).

We then calculated the interval in white keys for substitution events between the correct score notation note and the corresponding performance note. Black keypresses were not considered in the analysis.

In the analyses, we included the event types correct, small error (one-note errors that were not corrected), and large error (two-note errors that were not corrected). All errors larger than two-note errors were excluded. Moreover, we only included error and correct events that were preceded and followed by a correctly played note, which also excludes correct notes played before or after miss events. Each of the 97 notes included in the score notation of each piece was played 10 times in the course of the experiment, which allowed us to calculate the note accuracy for every note as the percentage of times the note was played correctly. Only notes that had an accuracy higher or equal to 40% were considered in the analysis, to exclude notes that were played systematically wrong. Additionally, we only included notes for which at least one error trial and one correct trial was included to avoid confounds of note selection.

##### Behavioral Dependent Variables

Two behavioral measures served as dependent variables, which possibly differed between event types (correct, small error, large error). To investigate potential behavioral effects of error severity, namely on keypress volume and post-event slowing, the behavioral dependent variables Volume and Inter-Keypress-Interval (IKI) were assessed. Volume was defined as the recorded velocity in the MIDI signal of each note. IKI was defined as the difference between note onset time of the current and of the following note (see Paas et al., 2021). This maps the time delay between the event (correct, small or large error) and the subsequent correct keypress and serves as a measure of post-event reaction time, which is used to calculate post-error-slowing.

##### Behavioral data statistical analysis

For all statistical analyses, if not stated differently, we conducted single-trial linear mixed models (LME) analyses in R (version 3.5.3) using the package lme4 (version 1.1-23). According to best practice (Meteyard and Davies, 2020), all models should include all within-subject main and interaction effects as random effects, if this is possible without leading to model fit errors. For all subsequently described analyses, we performed an iterative process: all within-subject main and interaction effects were first included as random factors. If this led to model fit errors (singular fit or overfitting), we tested which random effect led to this error and removed this from the model. As most of our models included only the main effect Event Type, for some models this factor is included as random effect factor and for others not, depending on the model fit.

We conducted LME analyses, calculating separate models for dependent variables IKI (post-event reaction time) and volume (velocity). As independent variable, we set the three-level factor Event Type (correct, small error, large error). Small error was set as baseline condition to determine both the difference between correct and (small) errors and between small and large errors. Consequently, we created the design matrix depicted in Table 1 based on simple coding. We included random intercepts and slopes for Event Type per participant into each model.

**Table 1:** Design matrix of the factor event type

	Small error	Correct	Large error
**Small error**	**0.66**	**−0.33**	**−0.33**
Correct	**−**0.33	0.66	**−**0.33
Large error	**−**0.33	**−**0.33	0.66

*Note.* The first line depicts the baseline condition.

With Cook’s Distance outlier detection (using the “influence” function of the package stats, version 4.02, in R) based on the calculated models (with a cutoff value of 4/(n-number of predictors-1)), we removed 4 participants from the IKI analysis (remaining *n* = 17, 17–34 years, *M* = 22.8 years, *SD* = 4.4 years, 9 women, 8 men) and two participants from the volume analysis (remaining *n* = 19, 17–34 years, *M* = 23.2 years, *SD* = 4.4 years, 11 women, 8 men). Subsequently, the models were recalculated with the new sample.

##### EEG data preprocessing

We recoded the EEG marker files offline by synchronizing the markers sent every five notes with the recorded MIDI data using MATLAB. The new markers were then written into new marker files, which were loaded into Brain Vision Analyzer (Brain Products, Munich, Germany). Subsequently, we applied a 0.5-Hz high-pass and 30-Hz low-pass filter to the data (as suggested by Luck, 2014). As participants read score notations while they played and were not prevented from looking down on their hand (both to obtain maximum ecological validity), vertical and horizontal eye movements occurred frequently during the experiment and the corresponding artefacts had to be removed from the EEG data. For this, we used the Gratton and Coles ocular correction algorithm (Gratton et al., 1983). The respective hEOG and vEOG channels were used as reference for eye artefact detection. The data were segmented into 900-ms–long epochs starting 300 ms before note onset. Subsequently, an automatic artifact rejection based on the signal from the electrodes of interest Fz, FCz, and Cz was performed. The artifact rejection removed all segments that included voltage steps larger than 50 μV/ms, for which the difference between highest and lowest amplitude was more than 100 μV, in which amplitudes were lower than −100 μV or higher than 100 μV, and for which activity was less than 0.1 μV. On average, 12.1 segments per participant were removed (0-146 segments, *SD* = 31.7 segments). This left enough segments per participant and condition for the following analyses (see also Table S3 in the Supplementary Material).

The interval between 300 and 200 ms before the event was used for baseline correction (for similar procedures in sequential task paradigms, see Herrojo Ruiz et al., 2009; Maidhof et al., 2013). Single-trial data as well as averages for each Event Type were exported per participant.

**Fig. 3:**
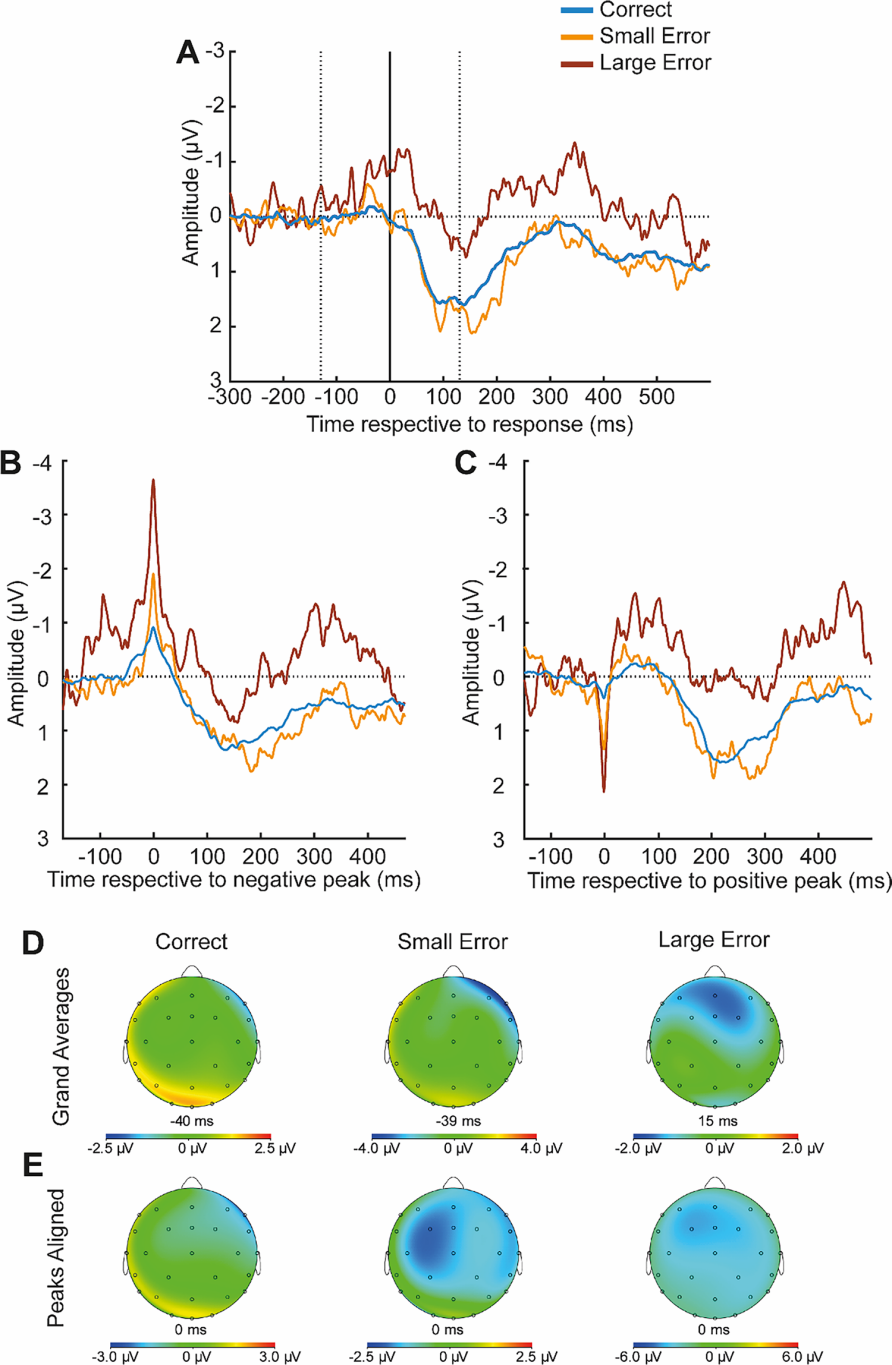
ERPs as a function of Event Type for Experiment 1. (**A**) ERPs respective to the response (correct keypress, small error or large error). (**B**) ERPs aligned for the negative peak latency identified for each participant and condition. (**C**) ERPs aligned for the respective preceding positive peak latency. (**D**) Topographies of the negative peak corresponding to the ERPs depicted in (A). (**E**) Topographies of the negative peak if the peaks are aligned (corresponding to the ERPs depicted in **B**)

In our statistical analysis of the ERN amplitude, we used ERP data from single trials. To determine the time points for data extraction in the single trials, we took the individual participants’ averages in each condition into account, thereby applying a combination of average- and single-trial-based analyses. Thus, only participants were included who had at least ten trials in each experimental condition. The EEG signal was first pooled at Fz, FCz, and Cz, because at these sites the ERN is typically maximally pronounced, which also was the case in the present study. Because participants were allowed to play in their individual tempo, and the latencies of ERNs in sequential tasks are related to movement onset (Maidhof et al., 2013) and thus indirectly to tempo, we expected large peak latency variations between participants which were visible in single-participant data inspection (for a display of single-subject ERPs, see Figure S5 in the Supplementary Material). To determine the typical ERN latencies in each participant, we considered the participants’ averages for each Event Type and searched for the maximum negative peak in in a time window between 130 ms pre- and 130 ms post-event. Likewise, we determined the latencies of the preceding maximum positive peak in a time window between 180 ms pre-event and the negative peak (for a similar procedure, see Maier et al., 2012). We subsequently calculated the single-trial amplitude measures corresponding to the peaks in the averages as the mean signal in the time window 10 ms before to 10 ms after the negative and positive peak latency in the average, respectively. Single-trial ERN measures corresponding to an average-based peak-to-peak measure were then calculated as the difference between the two values derived for each segment. We used difference measures (amplitude around negative peak – amplitude around positive peak), as segments might partly overlap in a sequential task and subtracting the preceding positivity can partially account for differences in baseline activity which can indeed be seen in Fig. 3. In two control analyses we used only single trial values corresponding to the negative peak in the average (without subtraction of the preceding positivity) or mean amplitude values in a time window from 50 ms before to 50 ms after keypress, because the relative negativity for errors was most pronounced in this time window. This analysis yielded comparable results (see Section S9 in the Supplementary Material).

##### EEG data statistical analyses

We defined an LME model with ERN amplitude as dependent variable (see above for the general procedure for defining LME models). Event Type served as independent variable, coded as in the behavioral analyses (Table 1). Random intercepts per participant were included (adding Event Type as random factor led to singular fit error). No participant was excluded based on Cook’s outlier detection.

### Results

Additional statistical results for all models can be found in the Supplementary Material (Tables S6, S7, and S8).

#### Expertise

Participants spent 7760.38 h on average playing the piano during their lifetime (range 520 h – 24,960 h, SD = 7,871.76 h, *Median* = 4,368.0 h; see Figure S4 for a histogram).

##### Behavioural data

On average, correct keypresses occurred in 90.70% of all keypresses, small errors in 3.03%, and large errors in 1.44%. All included participants made at least ten large errors. For detailed information, see Table S3.

##### IKI

There was a significant effect of Event Type on IKIs, *F*(2,15566) = 27.44, *p* < 0.001. Contrast comparisons revealed a significant difference between small and large errors (*p* = 0.001, *b* = 22.14), and between correct responses and small errors (*p* = 0.036, *b* = -4.71). After a large error keypress, participants took significantly longer (*M* = 369.66 ms, *SD* = 29.68 ms) to press the next key compared to after a small error keypress (*M* = 361.68 ms, *SD* = 16.30 ms), and the IKI after correct actions was shorter than after a small error keypress (*M* = 358.63 ms, *SD* = 4.99 ms).

##### Volume

We found a significant effect of Event Type, *F*(2,17509) = 88.23, *p* < 0.001. Both correct events (*p* < 0.001, *b* = 4.10) and large errors (*p* < 0.001, *b* = 3.96) resulted in significantly higher volume levels (*M* = 69.49 velocity, *SD* = 0.50 velocity; and *M* = 68.30 velocity, *SD* = 2.21 velocity, respectively) compared with small errors (*M* = 64.14 velocity, *SD* = 1.68 velocity).

##### ERN

For a display of ERPs in the three Event Type conditions, see Fig. 3 (Figure S5 in the Supplementary Material shows single subject averages). There was a significant effect of Event Type, *F*(2,2079.10) = 21.61, *p* < 0.001. Contrasts revealed significantly lower amplitudes for correct responses (*M* = −1.07 μV, *SD* = 0.34 μV) compared with small errors (*M* = −1.87 μV, *SD* = 0.96 μV; *p* < 0.001, *b* = 0.88) and significantly higher amplitudes for large errors (*M* = −3.16 μV, *SD* = 1.68 μV) compared with small errors (*p* = 0.004, *b* = −1.21). A significant difference between small and large errors remained if the single-trial ERN was quantified just based on the maximum negative peak in the average in a time window between –130 ms and 130 ms per participant and condition, and if the ERN was quantified as the amplitude mean between −50 ms and 50-ms relative to the button press, but no significant difference between small errors and correct events was found for these analyses (see Section S9 in the Supplementary Material).

### Conclusion for experiment 1

In Experiment 1, we compared the processing of different error types in a piano-playing paradigm. Our results show that ERN amplitudes as well as behavioral measures vary depending on the type of error. Larger ERN amplitudes were observed for large compared with small errors, whereas all errors were accompanied by a larger ERN relative to correct responses. Post-error-slowing was seen after all error types but was largest for large errors, whereas small errors were played in a lower volume than large errors and correct keypresses. The results indicate that the action monitoring system does not only differentiate between right and wrong but also between different degrees of erroneous actions. In a post-hoc analysis on measures that might represent expectancy, we found that error severity explained the effects better than the frequency of the event, the difficulty of the respective note, and the insecurity before and during the respective keypress (see Supplementary Material, Section S2).

## Experiment 2

Observing errors can be just as important as monitoring one’s own errors, for example, when musicians play together or teach others. As established above, the mechanisms of processing vicarious actions appear to be similar, albeit not completely identical, compared with those involved in the processing of own actions. Researchers observed a corresponding ERP component, the oERN (Bates et al., 2005; Miltner et al., 2004; van Schie et al., 2004), and increased activity in the mPFC for observed others’ errors (Koban and Pourtois, 2014).

As outlined for own responses above, also the neural response to observed actions can be modulated by surprise and expectancy (Alexander and Brown, 2011), as has been shown for mPFC activity (Schiffer et al., 2014) and the amplitude of a frontocentral oERN-like ERP component (Albrecht and Bellebaum, 2021a; Kobza and Bellebaum, 2013). Recent studies from our lab even suggest that previously observed valence effects for observed actions on this component can be completely attributed to expectancies (Albrecht and Bellebaum, 2021a, 2021b). The occurrence of an oERN-like component in action observation is contrary to the assumptions of the reinforcement learning theory (Holroyd und Coles, 2002), which assigns the ACC a role as motor control unit. In addition, the component seems to be primarily driven by expectancies, not valence, whereas the theory expects the signal to resemble a signed, rather than an unsigned, prediction error. Based on the empirical findings concerning the ERP component after observed actions, it is questionable whether the component is related to observed *error* processing at all. We will thus subsequently refer to it as observer mediofrontal negativity (oMN). The strong expectancy effect on the oMN amplitude may suggest a functional dissociation between ERN and oMN, with potentially differing effects of error severity on the two components.

As with active error processing, research on observed error processing has so far focused on a binary classification of response accuracy (Bates et al., 2005; de Bruijn and von Rhein, 2012; Kobza and Bellebaum, 2013). The observational data used in this study were taken from the actively performing participants of Experiment 1. We expected to see higher oMN amplitudes for errors than for correct keypresses, because errors were less frequent and thus more unexpected. Because there was only a slight difference between small and large error frequency in the videos for Experiment 2 and because we assumed that the oMN was mainly driven by the expectancy of the observed response, we suspected to find no difference in oMN amplitude between the error types and thus a different pattern as for own responses in Experiment 1.

To directly compare the processing of own and observed actions, we also conducted an analysis including the ERPs from experiments 1 and 2 with factors agency and event type. In this exploratory analysis, amplitude differences between the components ERN and oMN were eliminated via z-standardization. Because we hypothesized to find differences between small and large errors in the ERN, but not in the oMN, we expected to find a significant interaction between agency and event type.

### Method

#### Participants

As in Experiment 1, experienced pianists were recruited via print-material, social media, and mouth-to-mouth advertising. Again, a minimum experience of 1,500 h was suggested, but lower values were allowed if participants were able to play the respective material by heart (see below). Because sequential tasks have not been used for error observation paradigms before, we planned our sample size based on previous sequential task paradigms on own action monitoring (Herrojo-Ruiz et al., 2009; Kalfaoğlu et al., 2018; Maidhof et al., 2013; see also Experiment 1) and aimed for a final sample size of at least 20 participants. As in Experiment 1, we expected a 30% dropout rate and thus recruited 30 participants, of which one had to be excluded due to a previous neurological or psychological disease. We therefore recorded data from 29 observer participants. Of these, three had to be excluded due to technical problems and three others because of low performance in the pre- and post-performance test or during the experiment (see below). The remaining 23 participants consisted of 15 cis-gender men and 8 cis-gender women between 18 and 44 years (*M* = 24.5 years, *SD* = 6.4 years). One participant was left-handed, 22 right-handed (again, the left-handed participant performed as well as the other participants in the pre- and post-test). All participants reported no previous psychological or neurological illnesses, no intake of medication that could affect the nervous system, and had normal or corrected-to-normal vision. Participation was voluntary and participants received compensation of 40€ or course-credit. The study was in accordance with the declaration of Helsinki and approved by the ethics committee of the Faculty of Mathematics and Natural Sciences at Heinrich-Heine-University, Düsseldorf.

#### Material

Participants watched videos that were recorded during data acquisition of Experiment 1. In contrast to Experiment 1, participants were required to know the piece by heart to facilitate observation. To limit the time effort and ensure that participants reached a high performance level, we used only one of the six short pieces per participant that were used in Experiment 1. To obtain a large number of trials per condition, we calculated the number of isolated events for each event type and piece. Large errors were the most infrequent event type, so we chose the piece in which the most isolated large errors were made on average. Consequently, we chose 10 videos in which this piece was played (each from a different participant of Experiment 1) that included as many isolated pitch errors as possible and as few other error types as possible (e.g., missed notes, black key notes, pitch errors that deviated more than two white keys from the correct key). In total, participants watched the same piece being played 60 times. Originally, the intention was to play each of the ten videos six times. Due to a technical error, one of the ten chosen videos was watched 12 times, eight videos were watched six times each, and one video was not watched at all. As the order of the videos was randomized, however, and the focus of the study was on the processing of the single notes, we suspected that this technical error did not affect the results of the study, which was confirmed by a post-hoc analysis excluding trials from the video that was shown 12 times, which yielded the same pattern of results. Participants saw 6.600 isolated correct notes being played, 290 isolated small errors, and 210 isolated large errors (Table S10, Supplementary Material).

The expertise of the pianists that played the piece in the video ranged from 936 to 22,620 hours (*M* = 6,423.1 h, *SD* = 6,078.8 h). Videos had a resolution of 1,280*720 px and a framerate of 60. The videos always started 1 s (or 60 frames) before the first keypress and ended 1 s (or 60 frames) after the last. They were trimmed at the upper and lower side so that only the piano and the moving hand were visible. For practicing, participants received the score notation and auditory recording of the piece before the experiment.

#### Experimental task and setup

Similar to the procedure for Experiment 1, the material was sent to participants before testing, and they were instructed to practice approximately 15 minutes a day on average in a tempo that felt comfortable for them. In contrast to Experiment 1, they were, however, instructed to learn only one piece, and this by heart. Participants stated an average practice time of 130.0 minutes (*SD* = 93.1 minutes, range 44 – 420 minutes). Before the experimental observation task was conducted in the lab, participants were asked to perform the piece themselves on a digital piano (Casio LK-S450) while MIDI signal was recorded on a connected laptop. The piano was set on mute to avoid additional feedback and to make the conditions as similar as possible to Experiment 1.

**Fig. 4:**
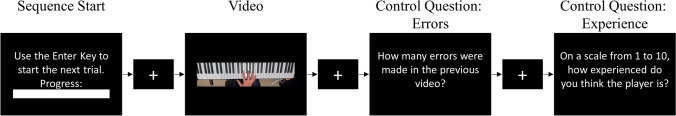
Sequence structure in Experiment 2

For the experimental task, participants sat in front of a 1,920 x 1,080 px desktop monitor. Participants were instructed to watch the videos carefully and count the errors made in each of them. The experiment consisted of 60 video presentations (9 different videos; durations between 31-70 s, *M* = 47.8 s, *SD* = 12.8 s), which were played in random order. The videos were embedded in sequences that also contained control questions after each video (see below). For a display of a sequence, see Fig. 4.

Participants could start the sequences themselves. After a short fixation cross (500 ms) the video was displayed. Participants received only visual input; the videos were played without sound. A marker was sent to the EEG recording software every fifth observed keypress. Following the videos and another 500-ms fixation cross, participants were asked how many mistakes the observed person had made in this segment. They could freely enter a number and proceed with the Enter key. After another 500-ms fixation cross, participants were asked how experienced in piano playing they believed the observed person to be on a scale from 1 to 10. Again, they could enter a number and proceed with the Enter key. Subsequently, the next sequence came on, which could again be started by the participant. Stimulus presentation and recording was controlled with Presentation (version 22.0, Neurobehavioral Systems, Albany, CA). After completing the experiment, participants were again asked to play the piece on the muted digital piano while MIDI was recorded.

##### Assessment of expertise

We acquired the measure Piano Playing Expertise in Experiment 2 in the same way as in Experiment 1.

#### Procedure

Participants received the material to practice the piano pieces used in the experiment via e-mail 2 weeks before the actual study in the lab. For testing in the laboratory, participants first gave written, informed consent to take part in the study. After this, they played the studied piece by heart. Participants subsequently filled out the demographic questionnaire, including Expertise measurements, after which EEG electrodes were attached. Participants then completed the actual experiment which lasted around 60 minutes. Finally, the electrodes were removed, and participants played the piece again. Participants received either course credit or 40 € as compensation.

#### EEG recording

EEG measures were recorded in the same way as in Experiment 1. Markers were sent and reconstructed in the same way.

#### Data analyses

##### Behavioral data of the pre- and post-tests

All following steps were performed in MATLAB, version R2017b (Mathworks, Natick, MA). As for Experiment 1, we used the dynamic score matcher algorithm created by Large (1993; see also Palmer and van de Sande, 1993; Rankin et al., 2009) to compare the recorded MIDI signal with the correct score notation for the pre- and post-experiment piano performance. We calculated the accuracy as the percentage of correctly played notes for each participant, separately for the pre- and post-experiment piano performance. If participants restarted playing the piece during the recording, all previous notes were excluded from further analysis. All participants who had an accuracy of <50% in both tests were excluded. This was the case for two participants in total.

##### Event types used for the ERP analysis

We used the same, previously determined relevant notes and events from Experiment 1 for the ERP analysis in Experiment 2. For this purpose, the notes and event types were extracted from the logfiles corresponding to the respective videos shown in Experiment 2. Inclusion criteria for notes were identical to the Experiment 1 analyses, with the exception that we also included notes with <30% note accuracy in Experiment 2. High error rates might indicate systematic errors for the players themselves but do not indicate potential systematic errors of the observer participants. A computer error during testing caused some videos to end too early for seven participants. In only one of them this led to a significant decrease in analyzable segments, and this participant was thus excluded from the analysis (this is one of the exclusions due to technical problems mentioned in the Participant section).

##### Behavioral data assessed during the experiment and data extracted from the participants of experiment 1

We assessed the measure Number of Perceived Errors (as stated by the participants after each sequence) and then calculated the measure Recognized Error Margin as the absolute difference between the Number of Perceived Errors and the actual error number (as calculated from the logfiles of Experiment 1; all error types were included in this measure). One participant of Experiment 2 who scored more than 1.645 *SD* higher (equivalent to a percent rank < 5) than the other participants in the Recognized Error Margin was excluded from all further analyses. Subsequently, the Perceived Expertise of the observed player (as stated by participants after each sequence, see above) and Objective Expertise of the observed player (Expertise measurement calculated for each player from Experiment 1) were determined. All continuous measures that were considered subsequently as factors in any analysis were scaled to lie between −0.5 and 0. 5 and then mean-centered.

##### Behavioral data statistical analysis

For the analysis of the behavioral data of the pre- and post-test, an LME analysis in R (version 3.5.3) was performed with accuracy as dependent variable and Measurement Time as fixed-effect factor. Random intercepts per participants were allowed. For the procedure determining the final model in terms of the random effects structure, please refer to the *Methods* section of Experiment 1.

Additionally, we investigated the relationship between perceived expertise and objective expertise of the observed player. An LME model with perceived expertise as dependent variable and Objective Expertise as fixed effect was defined, which allowed random intercepts and slopes for Objective Expertise by participant and random intercepts by observed video. Then, it was examined whether adding the Number of Perceived Errors (as stated by participants after each trial) in the respective trial explained significantly more variance by using model comparison. If the variable explained more variance, it was added to the model.

##### EEG data preprocessing

First, EEG markers were recoded based on the MIDI data gained in Experiment 1 for each of the observed players by using MATLAB. Subsequently, the markers were imported to Brain Vision Analyzer (Brain Products, Munich, Germany) for EEG data preprocessing, which was conducted in the same way as the processing described for Experiment 1. The artefact rejection removed an average of 5.2 segments (range 0-86 segments, *SD* = 17.0 segments).

Segments were also created in accordance with the procedure in Experiment 1, resulting in three Observed Event Types: observed correct response, observed small error, and observed large error. Again, single-trial data and averages per Observed Event Type and participant were exported and electrodes Fz, FCz, and Cz were pooled.

**Fig. 5:**
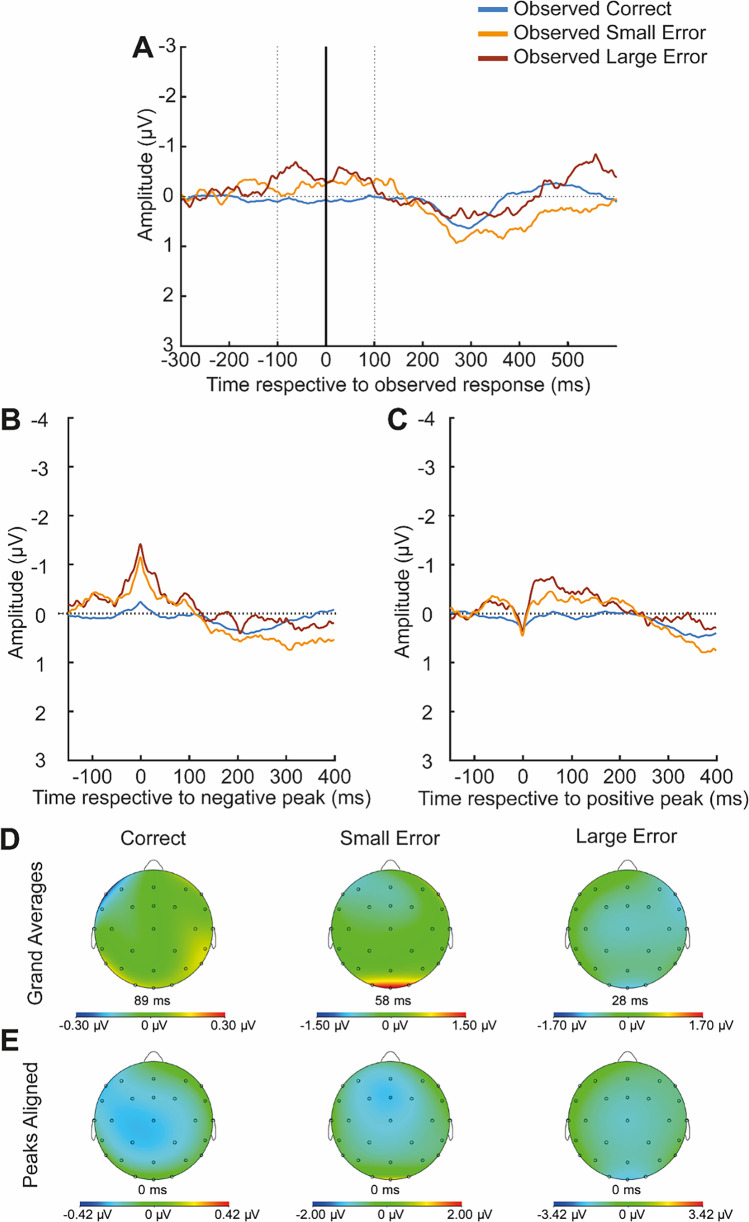
ERPs of observers as a function of Observed Event Type (Experiment 2). (**A**) ERPs respective to the observed response (observed correct keypress, observed small error or observed large error). (**B**) ERPs aligned for the negative peak identified for each observer participant and condition. (**C**) ERPs aligned for the respective preceding positive peak. (**D**) Topographies of the negative peak corresponding to the ERPs depicted in Figure **A**. (**E**) displays the topographies of the negative peak if the peaks are aligned (corresponding to the ERPs depicted in **B**)

The component that we call oMN (often referred to as oERN in the literature) occurs later than the ERN in nonsequential tasks: namely 100 to 300 ms after the event (depending on the task, see Bates et al., 2005; Miltner et al., 2004; van Schie et al., 2004). To date, this component has not been investigated in sequential tasks. If, however, earlier ERN peaks in active sequential tasks are related to the earlier onset of the movement relative to key registration compared with nonsequential tasks (Di Gregorio et al., 2022; Maidhof et al., 2013), it is conceivable that the oMN also peaks earlier in sequential tasks, as the observed movement can be detected earlier. Indeed, visual inspection of our data revealed a negativity that seemed to represent action monitoring between −100 and 100 ms around the observed keypress (see Fig. 5 for a grand average, and Figure S13 in the Supplementary Material for single subject ERPs). In accordance with this, we again used a combination of average-based and single-trial based analyses for the extraction of the ERP signal of interest. First, we determined the latencies of the maximum negative peak in a time window between −100 ms pre-event and 100 ms post-event in the average of each participant in each Event Type condition. Then, the preceding positive peak was searched in the time window between −150 ms and the negative peak. Again, single-trial measures corresponding to these peaks in the average were calculated in an area from 10 ms before to 10 ms after the negative and positive peak latency for the respective participant and condition, and measures corresponding to an average-based, peak-to-peak measure for each trial were determined as the difference between the two values. Because peaks in the average were not as pronounced as in the active data, and latencies might have varied both between participants and between trials within participants (due to different playing speeds of the different players in the videos), we conducted additional analyses based on just the amplitude value corresponding to the negative peak and based on the mean amplitude in the time window from −100 to +100 ms with the same model. Compared with the data of active responders (Experiment 1), the relative negativity was temporally more dispersed around the onset of the observed button press. That’s why a wider time window was used for the mean amplitude-based analysis. Results are reported in the Supplementary Material. Both analyses led to a similar result pattern than the main analysis. As for Experiment 1, ten trials were required as minimum for each participant in each condition, which was fulfilled by all participants of Experiment 2.

No subject was removed as outlier for the oMN analysis based on Cook’s Distance. After data-cleaning was complete, more than 100 segments remained for each participant and condition (Table S11).

##### EEG data statistical analyses

We first defined an LME model with oMN amplitudes as dependent variable and Observed Event Type as independent variable, coded as in the previously described analyses for Experiment 1 (Table 1). Random intercepts per participant were set (see *Methods* for Experiment 1 for the procedure in determining the random effects structure of the model). All continuous measures that were considered as predictors subsequently were scaled to lie between −0.5 and 0.5 and then mean-centered.

We additionally tested whether adding the Recognized Error Margin, which represented error recognition accuracy and may be affected by attentional factors, as independent variable explained more variance.

##### Post-hoc analyses comparing active and passive ERP data

In an exploratory post-hoc analyses, we compared the effects of the factor Event Type between the ERN and oMN, that is, between the active and observer participants of Experiments 1 and 2. As we were not interested in amplitude differences between the two dependent ERP variables (ERN and oMN), we used z-transformed data of both the active and observer groups. We determined a model with Event Type (correct, small error, large error, coded as in the previous analyses) as within-subject and Agency (active, observer, coded as −0.5 and 0.5, respectively) as between-subject fixed effect factor. Z-standardized single trial ERP amplitudes were set as dependent variables. Random intercepts per participant were allowed. A potential interaction was resolved by determining the Agency effect for the respective conditions correct, small error and large error.

### Results

Additional statistical results for all models can be found in the Supplementary Material (Tables S14, S15, and S16).

#### Expertise

Participants had a mean Expertise of 4,913.09 hours (*SD* = 4,404.90 h, 780-1,7160 h, *Median* = 3,432.0 h). Although means and medians were descriptively different, Expertise did not differ significantly between the samples of Experiments 1 and 2 (*t*(42) = 1.50, *p* = 0.141), possibly due to the high variance of Expertise in both groups. Previous studies suggest that changes in action monitoring occur already at early stages of training (Jentzsch et al., 2014; Rachaveti et al., 2020). Whereas all of our participants were highly experienced, we can assume that the descriptive differences in Expertise between groups did not influence results. For a histogram of expertise, see Figure S12 in the Supplementary Material.

#### Behavioral data of the pre- and post-test

Participants had an average accuracy of 86.21% in the pre-experimental test (*SD* = 11.88%), and an average accuracy of 83.80% in the post-experimental test (*SD* = 16.18%), which did not differ significantly *F*(1,22.00) = 1.18, *p* = 0.289, *b* = −2.42. This indicates that participants did not learn additionally by watching the 60 repetitions of the piece.

#### Behavioral data assessed during the experimental task

Regarding the Recognized Error Margin, participants differed on average by 5.60 (*SD* = 1.66) perceived errors from the actual errors in the videos. The Recognized Error Margin was calculated as the absolute difference in each sequence between the number of actual and recognized errors. Looking at over- and underestimation separately, participants underestimated the number of errors in 76.73% of trials on average (*SD* = 20.85%), overestimated the number of errors in 15.39% on average (*SD* = 18.40%) and correctly stated the number of errors in 7.88% on average (*SD* = 4.71%). In sequences in which participants underestimated or correctly estimated the number of errors, they failed to notice an average of 44.98% of errors (*SD* = 14.15%). In sequences in which participants over- or correctly estimated the number of errors, they noticed on average 46.39% of additional errors (*SD* = 12.73%). The results show that participants were not excellent at recognizing errors. However, the variance between participants was relatively small. To control for interindividual differences regarding error recognition, we considered the Recognized Error Margin as a variable in our main ERP analysis.

In a model including perceived expertise as dependent and Actual Expertise as independent variable, adding the perceived number of errors led to a significantly improved model fit, χ^2^(2) = 174.30, *p* < 0.001, *AIC*_without_ = 4,859.10, *AIC*_with_ = 4,688.80. The actual expertise of the players did not influence the perceived expertise as a main effect (*p* = 0.952) or in interaction with the Perceived Number of Errors (*p* = 0.071), but we found a main effect of Perceived Number of Errors, *F*(1,1307.63) = 186.00, *p* < 0.001, *b* = −6.23. A higher number of perceived errors led to lower perceived expertise.

#### EEG data

ERPs in response to the different Observed Event Types are displayed in Fig. 5 (for single-subject ERPs, see Supplementary Material, Figure S13). For the number of segments included in each condition for the analysis, see Table S11. Adding the Perceived Number of Errors (*p* = 0.780) explained no additional variance compared to a model with Observed Event Type as the only predictor. We found a main effect of Observed Event Type on oMN amplitude, *F*(2,21158.00) = 24.56, *p* < 0.001. The contrast between observed small errors (*M* = −1.19 μV, *SD* = 0.52 μV) and observed correct keypresses (*M* = −0.44, *SD* = 0.17 μV; *p* < 0.001, *b* = 0.73) revealed a significant difference, but no difference was found between large (*M* = −1.35 μV, *SD* = 0.60 μV) and small errors (*p* = 0.480, *b* = −0.15). Calculating the oMN as a mean amplitude between −100 and 100 ms around the observed response or using the amplitude corresponding to the maximum negative peak in the average in the respective timeframe revealed a similar pattern (see Supplementary Material, Section S17).

#### Exploratory analysis comparing active and observer ERP data from experiments 1 and 2.

Although there was a main effect of Event Type, *F*(2,61471.00) = 43.77, *p* < 0.001, and a trend effect of Agency, *F*(1,172.00) = 3.43, *p* = 0.066, our main interest was the interaction between Agency and Event Type. This interaction was significant, *F*(2,61471.00) = 3.01, *p* = 0.049. Resolving the interaction, there was no effect of Agency for correct events (*p* = 0.855) or small errors (*p* = 0.983), but for large errors *F*(1,3693.00) = 6.15, *p* = 0.013, *b* = 0.11, the standardized amplitudes of the ERP component related to monitoring were significantly larger for the active compared with the observer group.

### Conclusion for experiment 2

We studied the processing of different error types in an action observation paradigm in which participants watched videos of others playing the piano. In accordance with previous studies we found larger amplitudes of the oMN for errors versus correct responses. As in most studies investigating (observed) error processing (e.g., Miltner et al., 2004; van Schie et al., 2004), valence effects in this study are confounded by low frequencies of errors. In accordance, a post-hoc analysis (see Section S2 in the Supplementary Material) revealed that between-condition differences in observed event type frequencies could explain the result pattern as well as differences in observed action valence. The focus of the study was, however, on potential effects of error severity, and there was no significant difference between observed small and large errors. The result pattern thus differed from the one in Experiment 1, where we found error severity effects for own action processing. The difference between active and observational response monitoring was further supported by an exploratory analysis directly comparing the data obtained in both experiments. This analysis indeed revealed that large errors, but not small errors and correct responses, were processed differently between active and observer participants, indicating that the error type is less influential in observed action processing than in own action processing.

## General discussion

Experiment 1 was designed to identify the effect of error severity on behavioral and electrophysiological action monitoring during piano playing. In Experiment 2, we investigated the electrophysiological effect of error severity when observer participants watched videos of pianists playing.

In line with the hypothesis, Experiment 1 revealed increased ERN amplitudes for large compared with small errors, which, in turn, elicited larger ERN amplitudes than correct responses. Previous research also found a distinction between different error types (Bernstein et al., 1995; Maier et al., 2008; Maier et al., 2012; Maier and Steinhauser, 2016). However, our study is the first to directly test the effect of error severity within one action dimension. Behaviorally, participants showed larger post-error slowing for large than for small errors. Overall, we thus found clear effects of error severity. Post-error slowing was reported in some previous studies investigating piano play (Herrojo Ruiz et al., 2009; Paas et al., 2021). The small post-error slowing after small errors (especially compared to post-error slowing after large errors) might be attributed to the expertise in our sample: some previous studies showed that expertise reduced or even eliminated post-error slowing (Crump and Logan, 2013; Jentzsch et al., 2014; Loehr et al., 2013; Rachaveti et al., 2020), depending on task demands (Jentzsch et al., 2014). Furthermore, if speed—or keeping a respective tempo—was emphasized, post-error slowing was reduced or not present (Jentzsch and Leuthold, 2006; Loehr et al., 2013). In the present study, participants had to keep the tempo, possibly explaining why little slowing occurred after small errors. Large errors, on the other hand, might have posed more demands with respect to corrective movements and attention, leading to the observed large post-error-slowing, possibly due to a reorienting process (Buzzell et al., 2017; Notebaert et al., 2009; Núñez Castellar et al., 2010).

As found in previous studies, participants played small errors significantly more quietly than correct notes (Herrojo Ruiz et al., 2009; Maidhof et al., 2009; Maidhof et al., 2013; Paas et al., 2021), whereas large errors were played at a similar volume as correct notes. We included only notes that were succeeded by a correct keypress, so for all (“uncorrected”) errors, hand movements following the error had to be adapted to keep on playing successfully (sequential correction). Together with the finding of post-error slowing, the reduced volume for only small errors suggests that action correction for small errors might start earlier than for large errors (even at keypress), which confirms recent findings on early error movement cancellation effects (Foerster et al., 2022).

For action observation (Experiment 2), there was no difference in processing between small and large errors. However, in accordance with previous studies, a significantly larger oMN amplitude for observed errors compared with correct keypresses was found (Bates et al., 2005; Bellebaum et al., 2020; de Bruijn and von Rhein, 2012; Koban et al., 2010; Miltner et al., 2004; van Schie et al., 2004). Error recognition accuracy, that is, the difference between the number of perceived and actual errors, did not explain additional variance in the model. Thus, even though the null effect does not allow the conclusion that error severity does not affect observed action processing, we assume that the effect is at least reduced in comparison to own errors. This assumption was further supported by an exploratory analysis in which we compared the ERP amplitude pattern between the active (Experiment 1) and observer (Experiment 2) groups. We found that only for large errors, z-standardized amplitude values were significantly larger for the active than for the observer group.

The different findings in action monitoring for action and observation might have theoretical implications. The PRO model states that mPFC activity reflects the (un)expectedness of outcomes and actions rather than their accuracy (Alexander and Brown, 2011; Gawlowska et al., 2018; Kobza and Bellebaum, 2013; Schiffer et al., 2014; Wessel et al., 2012). In contrast, the reinforcement learning theory (Holroyd and Coles, 2002) assumes that the ERN reflects a reinforcement learning signal that the ACC uses to adapt motor activity based on valence and expectancy of the event. Our data suggest that the monitoring of own actions at least partially reflects the deviation from a (subjective) goal, in line with the reinforcement learning theory (Holroyd and Coles, 2002). For observed action monitoring, however, the results might not reflect this deviation, but possibly only an expectancy violation (see also the effects of Event Type Frequency on both active and observation condition reported in the Supplementary Material, section S2). These results, in contrast, are more in line with the PRO model (Alexander and Brown, 2011). We suggest an integration of the two models: Based on the finding of different activations depending on the error size, we assume that the action monitoring system sends a general need-to-adapt signal to update action models (as proposed by the reinforcement learning theory) as well as prediction models (as proposed by the PRO model). For predictions, the magnitude of the adapt-signal depends on the prediction error, which has been shown for different event types: For feedback processing, for example, larger ERP amplitudes were found for infrequent compared to frequent feedback, irrespective of feedback valence (Ferdinand et al., 2012). Also, prediction error size modulates trial-by-trial ERP amplitudes in feedback processing (Fischer and Ullsperger, 2013; Ullsperger et al., 2014), suggesting that amplitudes depend on the size of the prediction adaptation. However, the two aforementioned studies showed modulations of a signed prediction error; thus, any effects can be accounted for not only by expectancies, but also by valence, and valence does seem to play an important role in feedback processing (Proudfit, 2015). We observed a similar effect of prediction error size for the processing of others’ actions, when less predicted actions elicited larger oMN amplitudes, irrespective of action valence (Albrecht and Bellebaum, 2021b). We believe that the adapt-signal, or maybe two overlapping adapt-signals, code the magnitude of prediction (Albrecht and Bellebaum, 2021b; Ferdinand et al., 2012) and action adaption needed to meet the desired outcome (as in the current study) continuously (rather than dichotomously). This combination of the reinforcement learning theory and PRO model could explain the magnitude of adapt-signals for cases where either an action or a prediction model or both have to be updated. Whether action or prediction adaptations are needed highly depends on the task: in observation, if others’ movements cannot be influenced (as in our study), an adapt-signal should be sent for predictions, but in active performance, especially in a sequential task, the adapt-signal should (also) be highly dependent on the necessity to update action models.

Future studies might test this suggested combination of the two models for both own and observed actions by modulating the necessity to adapt movements quickly (sequential vs. nonsequential task) and, especially in observed action, the possibility to adapt actions at all (observation vs. joint-action tasks; Loehr et al., 2013; Paas et al., 2021). Additionally, the continuous, nondichotomous, nature of the signal should be tested by introducing multiple valence levels (correct, almost-error, small error, large error, etc.) and extending findings on multiple expectancy levels (from highly expected to highly unexpected, possibly by modulating both signed and unsigned prediction errors). To further corroborate the dissociation between expectancy and error severity, participants’ expectancy regarding the action should be assessed directly after each trial.

### Conclusions

Our results offer first evidence for a continuous error severity coding in the brain during active action processing. Crucially, our results suggest that this effect cannot be (only) attributed to expectancies, suggesting a reliance on a more general need-to-adapt signal in action processing. In contrast, error severity did not modulate observed action monitoring, which is in line with prediction error coding and updating predictions. The divergent findings between action and observation concerning the effect of error severity might hint at the representation of different continuous need-to-adapt signals in the mPFC, with different signals playing larger roles in action or observation, respectively. This suggested combination of the reinforcement learning theory (Holroyd and Coles, 2002) and the PRO model (Alexander and Brown, 2011) should be tested empirically by introducing multiple valence and (extending previous research, see Albrecht and Bellebaum, 2021b; Ferdinand et al., 2012) expectancy levels, and manipulating the importance of action adaptation in own and observed action monitoring.

## Supplementary Information

ESM 1 (DOCX 2426 kb)
