# Supplementary material for: Slip or fallacy? Effects of error severity on own and observed pitch error processing in pianists
Source: Cogn Affect Behav Neurosci. 2023 May 17;23(4):1076–94. doi: 10.3758/s13415-023-01097-1 (PMC10400674; doi:10.3758/s13415-023-01097-1)
Supplement: Supplementary file 1 — (DOCX 2426 kb) [file 13415_2023_1097_MOESM1_ESM.docx]

**Supplementary Material for the manuscript**

**Slip or Fallacy? Effects of Error Severity on Own and Observed Pitch Error Processing in Pianists**

Christine Albrecht^1*^ and Christian Bellebaum^1^

^1^ Institute of Experimental Psychology, Heinrich Heine University Düsseldorf, Germany

**Figure S1**

*Piano pieces used in Experiment 1*


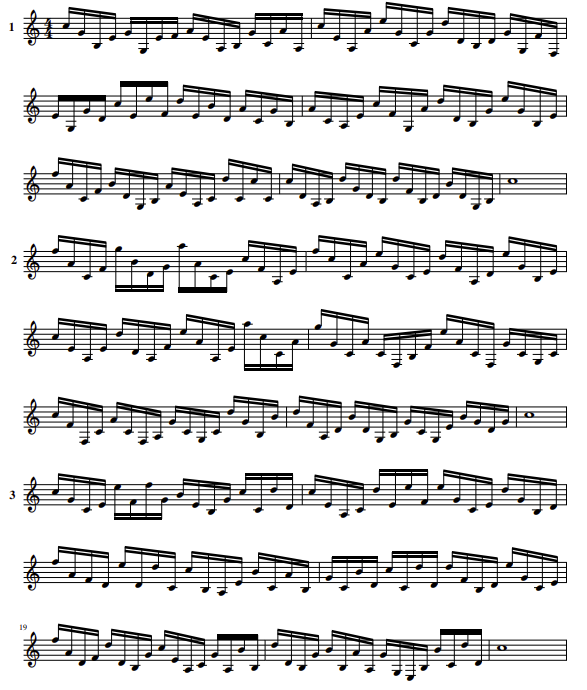


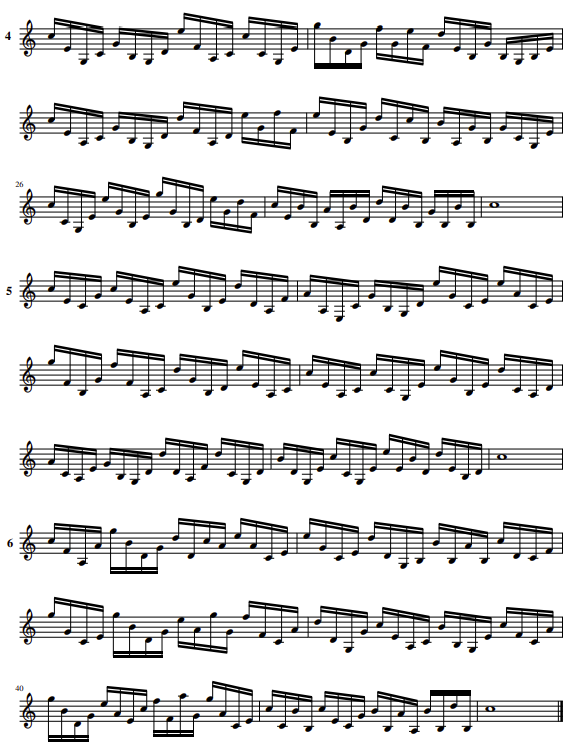


*Note.* In Experiment 2, only videos and material of piece 5 were used.

**Section S2**

*Effects of variables related to expectancies*

**Experiment 1.** We calculated four variables based on the behavioral data that could be related to participants’ expectancy regarding the action outcome and that were used as potential predictors of the dependent behavioral and ERP measures reflecting action monitoring. First, the inverted note accuracy (the non-inverted note accuracy was previously used as inclusion criterion), i.e. the note error rate, was defined as a measure for the difficulty of any included note. The higher the error rate, the more difficult the note should be for the respective participant, and the more errors they should expect (see Albrecht & Bellebaum, 2021b, for a modulation of expectancy by difficulty). Second, the distribution of event types was different for participants depending on their performance, and we expected that the more frequent an event type was, the more it would be expected. The percentage of all event types (correct, small error, and large error) for each participant formed the variable Event Type Frequency. Third, piano players often play either more loudly or more quietly in passages in which they are insecure, so changes in velocity can serve as an indicator of (in)security. The less secure participants are about a passage, the more they should expect to make errors, which is why insecurity might influence expectancies. Insecurity was calculated as the absolute difference between the velocity of each played note and the mean velocity for each participant. Fourth, we also calculated Insecurity of the previously played note to account for expectations building up before the actual keypress. All continuous measures that were considered subsequently in any analysis were scaled to lie between -0.5 and 0. 5 and then mean-centered.

**Experiment 2.** As measures that may affect expectancy of the observed response, we calculated the Difficulty of each note in the piece, i.e. the number of times in percent, that the note was played incorrectly across all 60 sequences, and the Observed Event Type Frequency, i.e. the number of times (in percent) that each event (correct response, small error, large error) occurred for each observed person. All continuous measures that were considered subsequently as factors in any analysis were scaled to lie between -0.5 and 0. 5 and then mean-centered.

***Statistical Analyses***

**Experiment 1.** We investigated potential effects of the Difficulty of the respective note, the Event Type Frequency for each participant, and the Insecurity with which participants played the current and previous note on IKIs and on the ERN (as the volume data were used to calculate one of the three potential predictors, we omitted this dependent variable from this analysis step). Additionally, we checked if these potential predictors of behavioral and ERP measures themselves differed by Event Type by calculating separate models with the respective variable as dependent variable and Event Type as independent variable (we allowed for random slopes and intercepts per participant). Then we checked whether either of the four potential predictors, when replacing Event Type as independent variable, led to a better model fit for predicting the behavioral IKI or the ERN. The models determined in the main analyses only containing Event Type as predictor were used for comparison. Again, we corrected the Alpha level with Bonferroni correction, this time to α = .013 due to the three model comparisons for each dependent variable.

**Experiment 2.** An analysis was conducted to test whether observed error processing (as reflected in the oMN) was better explained by either the Difficulty or the Observed Event Type Frequency of notes than by Observed Event Type. Additionally, we checked if a model involving the Perceived Expertise of the observed person explained significantly more variance than the model with only Observed Event Type as predictor.

***Results***

**Experiment 1.** Before examining potential effects of the four variables Event Type Frequency, Difficulty, Current Note Insecurity and Previous Note Insecurity on the behavioral and ERP measure of interest (see below), we checked whether Event Type affected the four variables. For this purpose, we calculated four models with the respective variable as dependent and the factor Event Type as independent variable. For *M* and *SD* of all three variables, see Table S4A. Event Type Frequency was significantly different between conditions, *F*(2,19571.00) = 671846.00, *p* < .001. Correct events were more frequent than small errors (*p* < .001, *b* = 81.53), and large and small errors also differed significantly (*p* < .001). Event Type also significantly affected Difficulty, *F*(2,19575.00) = 619.23, *p* < .001. Correct Events were associated with lower difficulty values compared to small errors (*p* < .001, *b* = -8.50), and large errors were associated with higher difficulty (*p* = .002, *b* = 1.69) compared to small errors. Note that this difference is descriptively small and statistical results are influenced by the high number of data points used. Current Note Insecurity values also differed significantly between conditions, *F*(2,19576.00) = 44.28, *p* < .001. In accordance with the results for the absolute volume levels (see above), both correct events and large errors led to less deviation from the mean volume than small errors (*p* < .001, *b* = -1.59 and *p* < .001, *b* = -2.51, respectively). Previous Note Insecurity did not differ as a function of Event Type (*p* = .123).

***IKI.*** The analysis of effects of Event Type Frequency, Difficulty Current Note Insecurity and Previous Note Insecurity on the IKI revealed that Difficulty predicted IKIs, *F*(1,14.10) = 6.24, *p* = .025, *b* = 17.97 (more difficult notes were followed by longer IKIs), as did Event Type Frequency, *F*(1,10.62) = 6.49, *p* = .028, *b* = -15.17 (lower frequencies led to longer IKIs) and Previous Note Insecurity, *F*(1,17.82) = 15.39, *p* = .001,b = 35.91 (higher insecurity led to longer IKIs), but Current Note Insecurity did not (*p* = .321). In the comparison between the main analysis model (Event Type as factor) and the models with either Difficulty, Event Type Frequency, Current Note Insecurity or Previous Note Insecurity as factor, Event Type as factor led to a better model fit (*AIC*_EventType_ = 182392) compared to Event Type Frequency, χ^2^(4) = 33.09, *p* < .001 (*AIC*_EventTypeFrequency_ = 182417), Current Note Insecurity, χ^2^(4) = 15.81, *p* = .003 (*AIC*_CNInsecurity_ = 182399) and Previous Note Insecurity χ^2^(4) = 53.23, *p* < .001 (*AIC*_PNInsecurity_ = 182437), but not Difficulty (*p* > .999, *AIC*_Difficulty_ = 182374),

***ERN.*** Event Type Frequency predicted ERN amplitudes, *F*(1,15418.00) = 36.51, *p* < .001, *b* = 1.42 (larger amplitudes for smaller frequencies), but Difficulty (*p* = .513), Current Note Insecurity (*p* = .054), and Previous Note Insecurity (*p* = .123) did not. The model with Event Type as a factor fitted the data significantly better (*AIC*_EventType_ = 145121) than a model with Difficulty, χ^2^(1) = 42.70, *p* < .001 (*AIC*_Difficulty_ = 145162), Event Type Frequency, χ^2^(1) = 6.80, *p* = .009 (*AIC*_EventTypeFrequency_ = 145126), Current Note Insecurity, χ^2^(1) = 39.41, *p* < .001 (*AIC*_CNInsecurity_ = 145159), or Previous Note Insecurity χ^2^(1) = 42.25, *p* < .001 (*AIC*_PNInsecurity_ = 145162).

**Experiment 2.** As all participants viewed the same videos (with small exceptions due to technical problems, see main manuscript), we did not calculate statistical differences between Observed Event Types concerning Observed Event Type Frequency or Difficulty. The mean values are displayed in Table S4B: observed correct keypresses were more frequent than both small and large errors, but small errors were only slightly more frequent than large errors. Observed correct keypresses were less difficult than both error types, but error types barely differed in their Difficulty. Both Observed Event Type Frequency, *F*(1,3378.30) = 47.40, *p* < .001, *b* = 0.91 (smaller amplitudes for more frequent events) and Difficulty, *F*(1,39113.00) = 22.81, *p* < .001, *b* = 1.08 (smaller amplitudes for easier notes) had a significant effect on amplitudes based on peak-to-peak measures in the average. However, only Observed Event Type Frequency, χ^2^(1) = 1.68, *p* = .195, *AIC*_EventType_ = 305798, *AIC*_EventTypeFrequency_ = 305798 served as a similarly good predictor as Observed Event Type. Difficulty was a significantly worse predictor, χ^2^(1) = 26.40, *p* < .001, *AIC*_Difficulty_ = 305822. Adding Perceived Expertise to the main analysis model (i.e. the model for the effect of Observed Event Type on oMN amplitude) did not explain additional variance (*p* = .072).

***Table S2A***

*Means and Standard Deviations for Difficulty, Event Type Frequency and Insecurity by Event Type for Experiment 1.*

| **Difficulty (in % errors)** | | |
| --- | --- | --- |
|  | *M* | *SD* |
| Correct | 20.05 | 0.44 |
| Small Error | 29.92 | 1.65 |
| Large Error | 30.72 | 2.82 |
| **Event Type Frequency (in % of all events)** | | |
|  | *M* | *SD* |
| Correct | 89.18 | 0.23 |
| Small Error | 5.91 | 0.36 |
| Large Error | 2.35 | 0.40 |
| **Current Note Insecurity (in velocity, absolute deviation from mean velocity)** | | |
|  | *M* | *SD* |
| Correct | 9.70 | 0.27 |
| Small Error | 11.59 | 0.97 |
| Large Error | 8.64 | 1.20 |
| **Previous Note Insecurity (in velocity, absolute deviation from mean velocity)** | | |
|  | *M* | *SD* |
| Correct | 9.04 | 0.26 |
| Small Error | 9.59 | 0.88 |
| Large Error | 9.32 | 1.21 |

***Table S2B***

*Means and Standard Deviations for Event Type Frequency and Difficulty by Observed Event Type for Experiment 2.*

| **Difficulty (in % correct)** | | |
| --- | --- | --- |
|  | *M* | *SD* |
| Correct | 19.12 | 0.27 |
| Small Error | 27.26 | 1.25 |
| Large Error | 27.89 | 1.27 |
| **Event Type Frequency (in % of all events)** | | |
|  | *M* | *SD* |
| Correct | 88.95 | 0.14 |
| Small Error | 7.10 | 0.32 |
| Large Error | 4.03 | 0.22 |

*Note.* M = Mean, SD = Standard Deviation. As all participants saw the same videos (except some minor variation due to technical errors), standard deviations are driven only by differences between videos (and, as for difficulty, differences between notes), but not by differences between participants.

**Table S3**

*Overall accuracy and frequency of included trials for the main analyses of Experiment 1*

| **Overall Accuracy** | | | | |
| --- | --- | --- | --- | --- |
|  | *M* | *SD* | *Min* | *Max* |
| Correct | 90.70 % | 6.07 % | 75.09 % | 97.44 % |
| Small Error | 3.03 % | 2.97 % | 0.42 % | 11.90 % |
| Large Error | 1.44 % | 1.66 % | 0.29 % | 7.64 % |
| Small Corrected Error | 0.97 % | 0.51 % | 0.07 % | 1.78 % |
| Other | 3.87 % | 2.40 % | 0.57 % | 9.88 % |
| Total | 6045.62 | 167.78 | 5778 | 6399 |
| Missing Notes | 30.29 | 36.84 | 0 | 143 |
| **Included Trials IKI** | | | | |
|  | *M* | *SD* | *Min* | *Max* |
| Correct | 792.94 | 235.65 | 411 | 1144 |
| Small Error | 92.47 | 62.75 | 14 | 229 |
| Large Error | 31.35 | 18 | 10 | 70 |
| **Included Trials Velocity** | | | | |
|  | *M* | *SD* | *Min* | *Max* |
| Correct | 795.11 | 228.14 | 411 | 1144 |
| Small Error | 92.47 | 67.03 | 14 | 229 |
| Large Error | 35.00 | 22.37 | 10 | 102 |
| **Included Trials EEG** | | | | |
|  | *M* | *SD* | *Min* | *Max* |
| Correct | 804.43 | 220.57 | 411 | 1144 |
| Small Error | 93.76 | 63.92 | 14 | 229 |
| Large Error | 34.86 | 22.29 | 10 | 102 |

**Figure S4**

*Distribution of Expertise across participants for Experiment 1*

**Figure S5**

*Single Subject ERPs as a function of Event Type for Experiment 1*


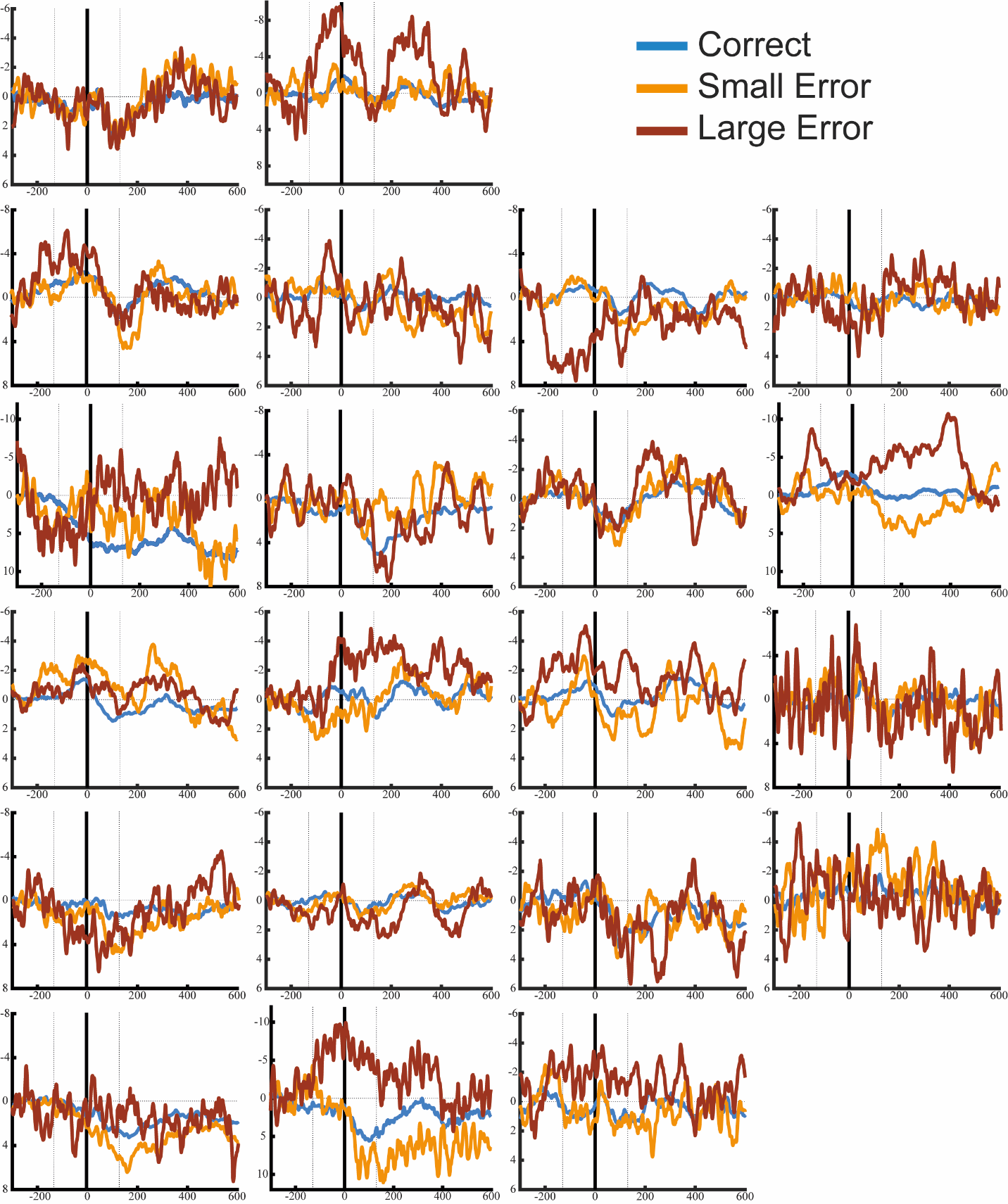


**Table S6**

*Statistical data for the IKI main analysis of Experiment 1*

| Effect | Estimate (*b*) | Std. error | *df* | *t*-value | *p*-value | CI 2.5% | CI 97.5% |
| --- | --- | --- | --- | --- | --- | --- | --- |
| Intercept (=Small Errors) | 370.20 | 25.81 | 16.08 | 14.34 | **<.001** | 323.42 | 420.51 |
| Correct | -4.71 | 2.25 | 15566.31 | -2.09 | **.036** | -9.18 | -0.39 |
| Large Errors | 22.14 | 4.23 | 15566.23 | 5.24 | **<.001** | 13.14 | 30.95 |

*Note*. Degrees of Freedom (*df*), *t*- and *p*-values as well as estimates (*b*) based on a restricted maximum likelihood approach, as proposed by Luke (2017) for the Event Type LME analysis on the IKI data. Satterthwaite approximation was used for the degrees of freedom. Significant values are displayed in bold font.

**Table S7**

*Statistical data for the volume main analysis of Experiment 1*

| Effect | Estimate (*b*) | Std. error | *df* | *t*-value | *p*-value | CI 2.5% | CI 97.5% |
| --- | --- | --- | --- | --- | --- | --- | --- |
| Intercept (=Small Errors) | 68.64 | 1.79 | 18.29 | 38.32 | **<.001** | 64.93 | 72.47 |
| Correct | 4.10 | 0.31 | 17510.00 | 13.27 | **<.001** | 3.55 | 4.77 |
| Large Errors | 3.96 | 0.56 | 17510.00 | 7.12 | **<.001** | 2.87 | 5.15 |

*Note*. Degrees of Freedom (*df*), *t*- and *p*-values as well as estimates (*b*) based on a restricted maximum likelihood approach, as proposed by Luke (2017) for the Event Type LME analysis on the volume data. Satterthwaite approximation was used for the degrees of freedom. Significant values are displayed in bold font.

**Table S8**

*Experiment1: Statistical data for the ERN analysis (quantified based on the peak-to-peak approach in the average)*

| Effect | Estimate (*b*) | Std. error | *df* | *t*-value | *p*-value | CI 2.5% | CI 97.5% |
| --- | --- | --- | --- | --- | --- | --- | --- |
| Intercept (=Small Errors) | -2.10 | 0.19 | 63.52 | -11.03 | **<.001** | -2.47 | -1.73 |
| Correct | 0.88 | 0.24 | 19436.39 | 3.76 | **<.001** | 0.41 | 1.36 |
| Large Errors | -1.21 | 0.43 | 19562.83 | -2.84 | **.004** | -1.98 | -0.38 |

*Note*. Degrees of Freedom (*df*), *t*- and *p*-values as well as estimates (*b*) based on a restricted maximum likelihood approach, as proposed by Luke (2017) for the Event Type LME analysis on ERN amplitudes. Satterthwaite approximation was used for the degrees of freedom. Significant values are displayed in bold font.

**Section S9**

***Section S9A***

*Experiment 1: Results for the ERN with a quantification based on only the negative peak in the average signal (see main text for details)*

In a model with Event Type as fixed factor, ERN amplitude as dependent variable and random intercepts per participant, there was a main effect of Event Type, F(2,19581.00) = 9.30, p < .001. Contrasts revealed no difference between ERNs for correct events (M = -0.74 µV, SD = 0.35 µV) and small errors (M = -0.96 µV, SD = 1.00 µV; p = .167, b = 0.33), but a significant difference between amplitudes for large (M = -2.27 µV, SD = 1.69 µV) and small errors (p = .004, b = -1.25), with larger amplitudes for large errors.

***Table S9B***

*Statistical data for the ERN with a quantification based only on the negative peak in the average*

| Effect | Estimate (*b*) | Std. error | *df* | *t*-value | *p*-value | CI 2.5% | CI 97.5% |
| --- | --- | --- | --- | --- | --- | --- | --- |
| Intercept (=Small Errors) | -1.40 | 0.23 | 38.56 | -6.00 | **<.001** | -1.90 | -0.92 |
| Correct | 0.33 | 0.24 | 19566.69 | 1.38 | .167 | -0.15 | 0.81 |
| Large Errors | -1.25 | 0.44 | 19590.28 | -2.87 | **.004** | -2.07 | -0.37 |

*Note*. Degrees of Freedom (*df*), *t*- and *p*-values as well as estimates (*b*) based on a restricted maximum likelihood approach, as proposed by Luke (2017) for the Event Type LME analysis on ERN amplitudes. Satterthwaite approximation was used for the degrees of freedom. Significant values are displayed in bold font.

***Section S9C***

*Results of the ERN main analysis using amplitude mean as ERN quantification*

In a model with Event Type as fixed factor, the amplitude mean between -50 and 50 ms as dependent variable and random intercepts per participant, there was a main effect of Event Type, *F*(2,19585.00) = 3.02, *p* = .049. Contrasts revealed no difference between amplitudes for correct events (*M* = -0.02 µV, *SD* = 0.32 µV) and amplitudes for small errors (*M* = 0.15 µV, *SD* = 0.89 µV; *p* = .625, *b* = -0.11), but a significant difference between amplitudes for large (*M* = -0.86 µV, *SD* = 1.48 µV) and small errors (*p* = .020, *b* = -0.94), with larger amplitudes for large errors.

***Table S9D***

*Experiment 1: Statistical data for the ERN with quantification as mean amplitude in a time window ± 50 ms around response onset.*

| Effect | Estimate (*b*) | Std. error | *df* | *t*-value | *p*-value | CI 2.5% | CI 97.5% |
| --- | --- | --- | --- | --- | --- | --- | --- |
| Intercept (=Small Errors) | -0.19 | 0.34 | 24.57 | -0.56 | .579 | -0.95 | 0.53 |
| Correct | -0.11 | 0.22 | 19588.28 | -0.49 | .625 | -0.52 | 0.34 |
| Large Errors | -0.94 | 0.40 | 19582.46 | -2.34 | **.020** | -1.69 | -0.12 |

*Note*. Degrees of Freedom (*df*), *t*- and *p*-values as well as estimates (*b*) based on a restricted maximum likelihood approach, as proposed by Luke (2017) for the Event Type LME analysis on ERN amplitudes. Satterthwaite approximation was used for the degrees of freedom. Significant values are displayed in bold font.

**Table S10**

*Distribution of Events in the 9 chosen videos in absolute numbers (Experiment 2)*

| Event Type | Position | *M* | *SD* | Minimum | Maximum |
| --- | --- | --- | --- | --- | --- |
| Correct | Total | 86.70 | 7.66 | 68 | 95 |
|  | Isolated | 66.00 | 13.37 | 41 | 85 |
| Error Not Corrected | Total | 5.20 | 4.08 | 1 | 13 |
|  | Isolated | 2.90 | 1.52 | 1 | 5 |
| Error Corrected | Total | 3.10 | 2.28 | 1 | 8 |
|  | Isolated | 2.10 | 1.20 | 1 | 4 |
| Error Large | Total | 1.78 | 0.67 | 1 | 3 |
|  | Isolated | 1.78 | 0.67 | 1 | 3 |
| Other events^a^ | Total | 2.30 | 1.64 | 0 | 5 |

*Note. M* = mean, *SD* = standard deviation. The video that was played twice as much as the other videos was included twice in calculations of mean and standard deviation.

^a^ other events comprise deleted notes, postslips, black keys, and errors that deviate more than 2 keys from the correct key. The piece consisted of 97 notes in total.

**Table S11**

*Included Trials in the observational ERP analysis (Experiment 2)*

| Event Type | *M* | *SD* | *Min* | *Max* |
| --- | --- | --- | --- | --- |
| Observed Correct | 1575.48 | 87.92 | 1261 | 1614 |
| Observed Small Error | 170.04 | 8.88 | 146 | 174 |
| Observed Large Error | 124.52 | 3.37 | 112 | 126 |

**Figure S12**

*Distribution of Expertise across participants for the observer dataset (Experiment 2)*

**Figure S13**

*Single Subject ERPs as a function of Event Type for Experiment 2*

**
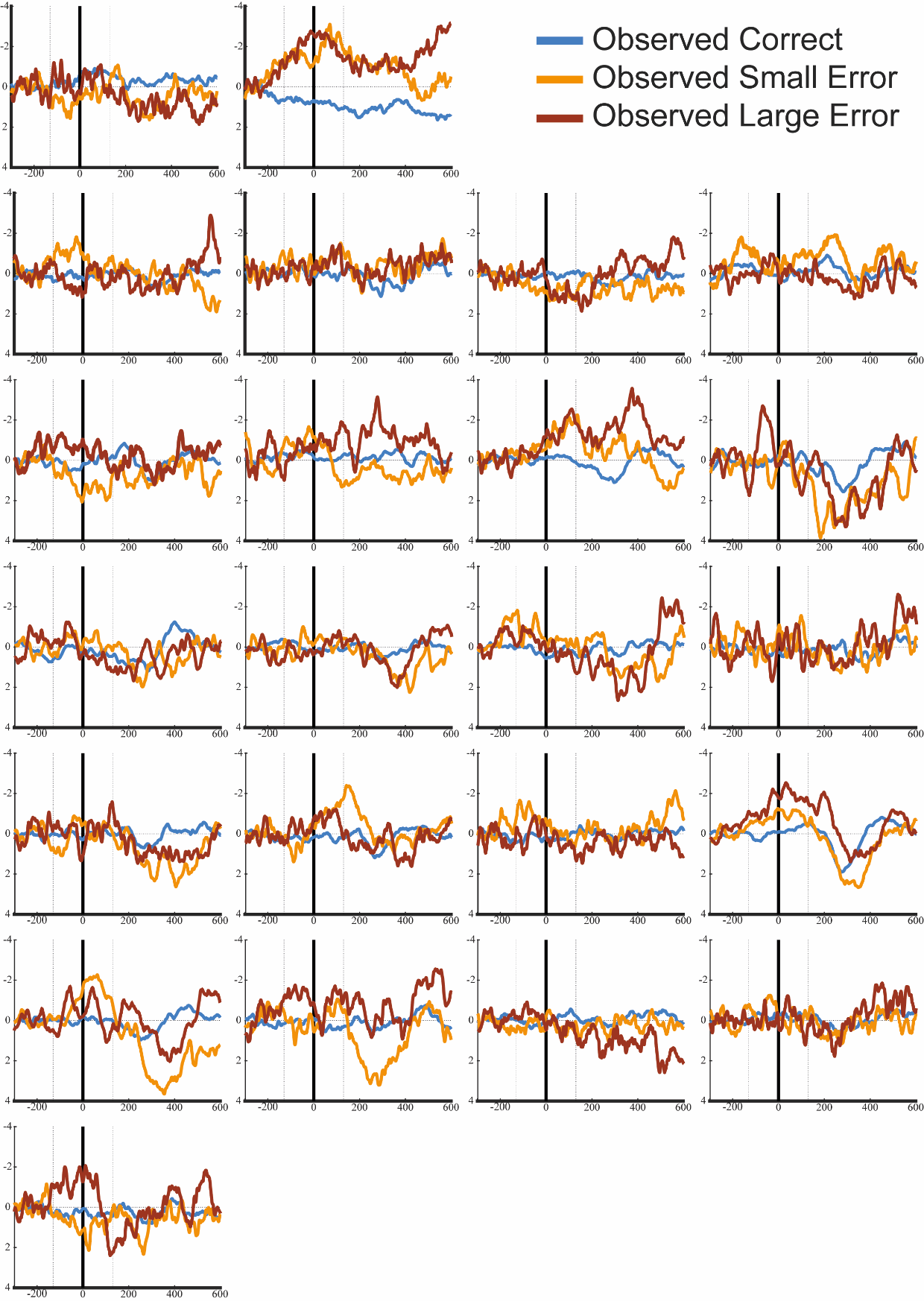
**

**Table S14**

*Statistical data for the pre- and post-test performance analysis comparing test times (Experiment 2)*

| Effect | Estimate (*b*) | Std. error | *df* | | *t*-value | *p*-value | CI 2.5% | CI 97.5% |
| --- | --- | --- | --- | --- | --- | --- | --- | --- |
| Intercept | 85.00 | 2.57 | | 22.00 | 33.11 | **<.001** | 80.27 | 90.19 |
| Test Time | -2.42 | 2.22 | | 22.00 | -1.09 | .289 | -6.46 | 1.76 |

*Note*. Degrees of Freedom (*df*), *t*- and *p*-values as well as estimates (*b*) based on a restricted maximum likelihood approach, as proposed by Luke (2017) for the Correction LME analysis on the ERN data. Satterthwaite approximation was used for the degrees of freedom. Significant values are displayed in bold font.

**Table S15**

*Statistical data for effect of Actual Expertise and Perceived Number of Errors on Perceived Expertise analysis (Experiment 2)*

| Effect | Estimate (*b*) | Std. error | *df* | | *t*-value | *p*-value | CI 2.5% | CI 97.5% |
| --- | --- | --- | --- | --- | --- | --- | --- | --- |
| Intercept | 5.77 | 0.34 | | 10.89 | 16.77 | **<.001** | 5.07 | 6.41 |
| Actual Expertise | 0.07 | 1.06 | | 7.53 | 0.06 | .952 | -2.20 | 2.11 |
| Perceived Number of Errors | -6.23 | 0.46 | | 1307.63 | -13.64 | **< .001** | -7.05 | -5.24 |
| Actual Expertise x Perceived Number of Errors | -3.46 | 1.92 | | 439.83 | -1.81 | .071 | -7.25 | 0.27 |

*Note*. Degrees of Freedom (*df*), *t*- and *p*-values as well as estimates (*b*) based on a restricted maximum likelihood approach, as proposed by Luke (2017) for the Correction LME analysis on the ERN data. Satterthwaite approximation was used for the degrees of freedom. Significant values are displayed in bold font.

**Table S16**

*Experiment 2: Statistical data for the oAM analysis (quantified based on the peak-to-peak approach in the average)*

| Effect | Estimate (*b*) | Std. error | *df* | *t*-value | *p*-value | CI 2.5% | CI 97.5% |
| --- | --- | --- | --- | --- | --- | --- | --- |
| Intercept (=Small Errors) | -0.97 | 0.09 | 20.31 | -10.36 | **<.001** | -1.15 | -0.77 |
| Correct | 0.73 | 0.14 | 19309.46 | 5.07 | **0**  **<.001** | 0.46 | 1.00 |
| Large Errors | -0.15 | 0.21 | 37983.92 | -0.71 | .480 | -0.54 | 0.29 |

*Note*. Degrees of Freedom (*df*), *t*- and *p*-values as well as estimates (*b*) based on a restricted maximum likelihood approach, as proposed by Luke (2017) for the Event Type LME analysis on ERN amplitudes. Satterthwaite approximation was used for the degrees of freedom. Significant values are displayed in bold font.

**Section S17**

***Section S17A***

*oMN amplitude quantified based on only the negative peak amplitude in the average (Experiment 2)*

When investigating the oMN amplitude quantified based only on the negative peak amplitude in each participant and condition found in the time window between -100 ms and 100 ms relative to the observed response, we again found no additional influence of Perceived Number of Errors (*p* = .256). There was a significant effect of Event Type on amplitudes, *F*(2,30837.00) = 28.08, *p* < .001, but contrast comparisons revealed a difference only between small errors and correct responses (*p* < .001, *b* = .79) and not between small and large errors (*p* = .262, *b* = -0.25). Descriptively, again, the largest negative amplitude was found for large errors (*M* = -1.19, *SD* = 0.65), followed by small errors (*M* = -0.94, *SD* = 0.59) and correct keypresses (*M* = -0.17, *SD* = 0.18). Additional statistical variables are displayed in Table S19D.

***Table S17B***

*Statistical data for the oMN analysis based only on the negative peak (Experiment 2)*

| Effect | Estimate (*b*) | Std. error | *df* | *t*-value | *p*-value | CI 2.5% | CI 97.5% |
| --- | --- | --- | --- | --- | --- | --- | --- |
| Intercept (=Small Errors) | -0.75 | 0.12 | 15.43 | -6.46 | **<.000** | -0.98 | -0.53 |
| Correct | 0.79 | 0.15 | 29286.99 | 5.16 | **<.000** | 0.51 | 1.12 |
| Large Errors | -0.25 | 0.22 | 41062.96 | -1.12 | .262 | -0.70 | 0.21 |

*Note*. Degrees of Freedom (*df*), *t*- and *p*-values as well as estimates (*b*) based on a restricted maximum likelihood approach, as proposed by Luke (2017) for the Event Type LME analysis on ERN amplitudes. Satterthwaite approximation

***Section S17C***

*oMN quantified as mean amplitude (Experiment 2)*

When investigating the oMN quantified as the mean amplitude between -100 and 100 ms we again found no additional influence of Perceived Number of Errors (*p* = .100). There was a significant effect of Event Type on amplitudes, *F*(2,36529.00) = 9.63, *p* < .001, but contrast comparisons revealed a difference only between small errors and correct responses (*p* = .012, *b* = .31) and not between small and large errors (*p* = . 188, *b* = -0.24). Descriptively, again, the most negative amplitudes were found for large errors (*M* = -0.45, *SD* = 0.53), followed by small errors (*M* = -0.23, *SD* = 0.46) and correct keypresses (*M* = 0.07, *SD* = 0.15). Additional statistical variables are displayed in Table S19D.

***Table S17D***

*Statistical data for the oMN mean amplitude analysis (Experiment 2)*

| Effect | Estimate (*b*) | Std. error | *df* | *t*-value | *p*-value | CI 2.5% | CI 97.5% |
| --- | --- | --- | --- | --- | --- | --- | --- |
| Intercept (=Small Errors) | -0.18 | 0.11 | 13.31 | -1.67 | .118 | -0.41 | 0.02 |
| Correct | 0.31 | 0.12 | 35531.88 | 2.50 | **.012** | 0.06 | 0.58 |
| Large Errors | -0.24 | 0.18 | 42146.03 | -1.32 | .188 | -0.61 | 0.11 |

*Note*. Degrees of Freedom (*df*), *t*- and *p*-values as well as estimates (*b*) based on a restricted maximum likelihood approach, as proposed by Luke (2017) for the Event Type LME analysis on ERN amplitudes. Satterthwaite approximation was used for the degrees of freedom. Significant values are displayed in bold font.

**Figure S18**

*Descriptive data for the comparison of active participants’ and observers’ z-standardized ERP data*


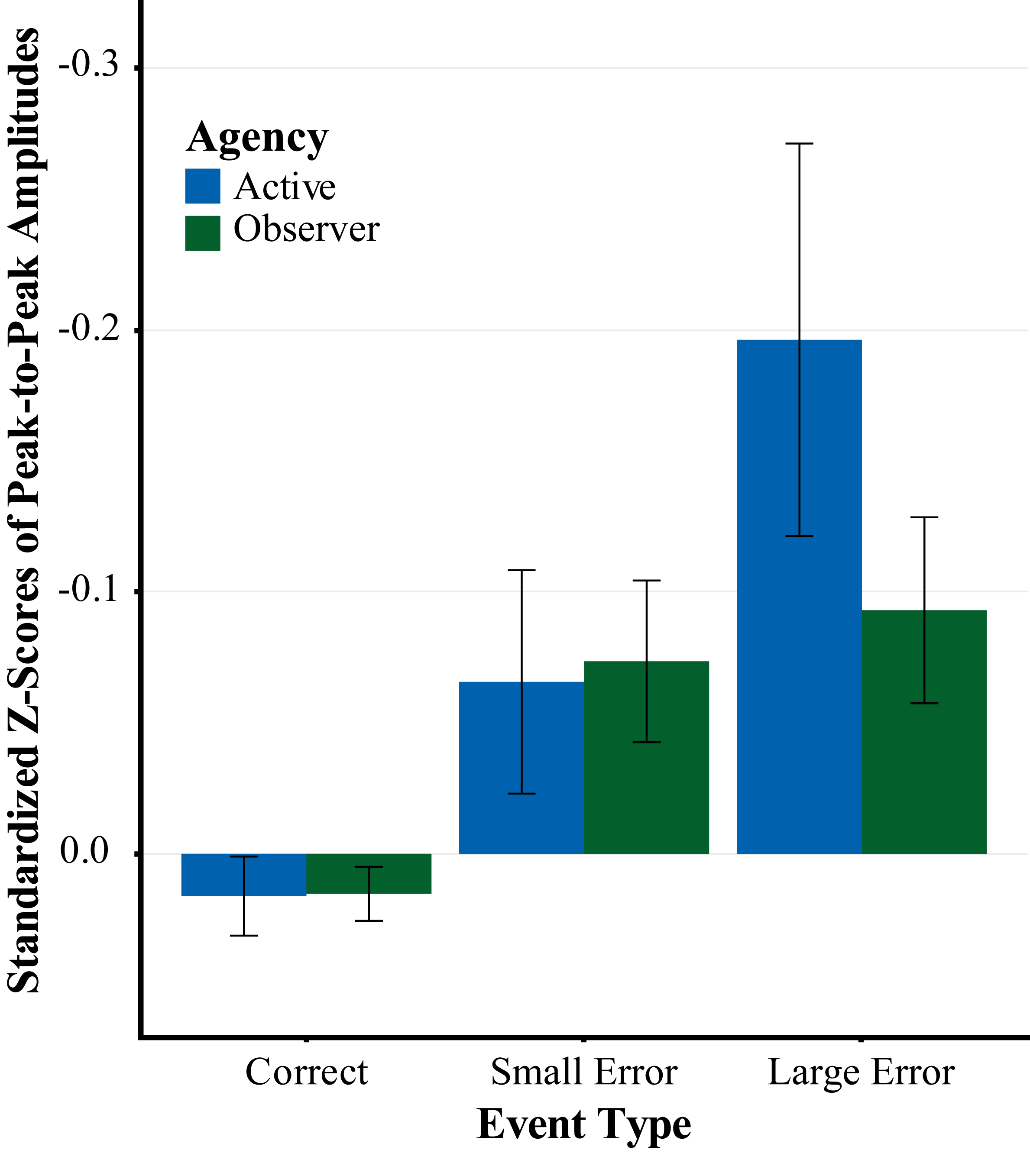


*Note.* Error bars represent confidence intervals.

**Table S19**

*Statistical data for the post-hoc analysis comparing active participants’ and observers’ ERP data*

| Effect | Estimate (*b*) | Std. error | *df* | | *t*-value | *p*-value | CI 2.5% | CI 97.5% |
| --- | --- | --- | --- | --- | --- | --- | --- | --- |
| Intercept (=Small Errors) | -0.07 | 0.01 | | 171.65 | -6.94 | <.001 | -0.09 | -0.05 |
| Correct | 0.09 | 0.01 | | 60895.77 | 6.04 | <.001 | 0.06 | 0.12 |
| Large Errors | -0.07 | 0.02 | | 61984.29 | -2.91 | .004 | -0.13 | -0.02 |
| Agency (Small Errors x Agency) | 0.04 | 0.02 | | 171.65 | 1.85 | .066 | 0.00 | 0.07 |
| Correct x Agency | 0 | 0.03 | | 60895.77 | 0.05 | .959 | -0.06 | 0.06 |
| Large Errors x Agency | 0.11 | 0.05 | | 61984.29 | 2.11 | .035 | 0.01 | 0.2 |

*Note*. Degrees of Freedom (*df*), *t*- and *p*-values as well as estimates (*b*) based on a restricted maximum likelihood approach, as proposed by Luke (2017) for the Correction LME analysis on the ERN data. Satterthwaite approximation was used for the degrees of freedom. Significant values are displayed in bold font.
